# Glucocorticoids in pregnancy: a master-switch for fetal maturation

**DOI:** 10.1210/endrev/bnag015

**Published:** 2026-05-23

**Authors:** Yoshi Tan, Michael J Stark, Vicki L Clifton, Michael D Wiese, Janna L Morrison, Kathryn L Gatford

**Affiliations:** Robinson Research Institute, College of Health, Adelaide University, Adelaide, SA 5005, Australia; School of Pharmacy and Biomedical Sciences, College of Health, Adelaide University, Adelaide, SA 5005, Australia; Early Origins of Adult Health Research Group, School of Pharmacy and Biomedical Sciences, College of Health, Adelaide University, Adelaide, SA 5005, Australia; Robinson Research Institute, College of Health, Adelaide University, Adelaide, SA 5005, Australia; Adelaide Medical School, Adelaide University, Adelaide, SA 5005, Australia; Pregnancy and Development, Mater Medical Research Institute, University of Queensland, Brisbane, QLD 4101, Australia; Centre for Pharmaceutical Innovation, Clinical and Health Sciences, Adelaide University, Adelaide, SA 5005, Australia; Robinson Research Institute, College of Health, Adelaide University, Adelaide, SA 5005, Australia; School of Pharmacy and Biomedical Sciences, College of Health, Adelaide University, Adelaide, SA 5005, Australia; Early Origins of Adult Health Research Group, School of Pharmacy and Biomedical Sciences, College of Health, Adelaide University, Adelaide, SA 5005, Australia; Robinson Research Institute, College of Health, Adelaide University, Adelaide, SA 5005, Australia; School of Pharmacy and Biomedical Sciences, College of Health, Adelaide University, Adelaide, SA 5005, Australia; Early Origins of Adult Health Research Group, School of Pharmacy and Biomedical Sciences, College of Health, Adelaide University, Adelaide, SA 5005, Australia

**Keywords:** pregnancy, corticosteroid, fetal development, respiratory system

## Abstract

The prepartum surge in plasma glucocorticoids is a critical signal for fetal organ maturation, facilitating the transition from fetal reliance on the placenta for functions such as respiration, nutrition, and waste removal to newborn use of their own physiological systems. The fetal glucocorticoid surge is conserved across mammalian species, but mechanisms driving the surge differ. Preterm birth interrupts fetal maturation because babies are born before the rise in endogenous glucocorticoids occurs, placing the newborn at greater risk of morbidity and mortality. In particular, lung immaturity increases risk of short-term and long-term respiratory disease. Antenatal corticosteroids (ACS) are routinely administered to women at risk of preterm birth to mimic the missed maturational signal of elevated glucocorticoids. Consequently, ACS improve neonatal survival and respiratory outcomes. Clinical guidelines universally recommend ACS use, but recommended regimens, gestational age limits, and use in settings of preexisting maternal conditions vary. Importantly, preterm infants have variable responsiveness to ACS treatment, and do not benefit equally. While male fetal sex and fetal growth restriction may be associated with lower ACS responsiveness, there is limited understanding of underlying factors. Emerging evidence points to differential glucocorticoid receptor isoform expression and elevated pretreatment plasma cortisol concentrations as potential contributors to variable ACS response. Improved insight into underlying mechanisms is crucial for identifying responsive subgroups and optimizing ACS treatment beyond the current one-size-fits-all approach to enhance health outcomes for preterm infants.

## Essential points

- The fetal glucocorticoid surge in late gestation is conserved across mammalian species with differences in underlying mechanisms
- Glucocorticoid receptors are highly expressed in many organs and their activation by glucocorticoid binding induces organ maturation in the fetus in preparation for the transition to postnatal function
- Antenatal corticosteroids are recommended in cases of threatened preterm birth, as they activate glucocorticoid signaling to promote lung maturation in preterm babies, reducing risk of respiratory distress syndrome and neonatal mortality
- Clinical guidelines for antenatal corticosteroid use vary across countries in relation to gestational age limits, timing of doses, and eligibility
- Preterm babies respond variably to antenatal corticosteroids, with poorer response associated with male fetal sex and fetal growth restriction, while underlying mechanisms remain poorly understood

Preterm birth, defined as the delivery of babies before 37 weeks of gestation (term = 40 weeks), is the leading cause of neonatal morbidity and mortality worldwide (1). An estimated 10% of babies globally are born preterm (2). Global preterm birth rates over the last decade have remained stable despite advances in pregnancy healthcare (1, 3, 4). This is concerning, as preterm infants miss critical signals that occur during late gestation, resulting in immature organs that are unprepared for ex utero life and increased risk of later behavioral, mental, and physical disorders (5-8). Respiratory distress syndrome (RDS) is the most common complication in preterm babies, affecting 45% of preterm infants (9), whose incomplete lung development results in poor capacity for gas exchange. One of the primary endogenous signals for organ maturation is a physiological rise in fetal plasma concentrations of glucocorticoids, steroid hormones that can bind the glucocorticoid receptor (GR), which is near-ubiquitously expressed in fetal tissues (10). Antenatal corticosteroids (ACS), synthetic glucocorticoids that readily cross the placenta when administered to the mother, can mimic these maturational effects. International clinical guidelines recommend maternal administration of a single course of ACS (24 mg of betamethasone or dexamethasone), given as multiple doses over 24 or 48 hours, when preterm birth is expected within a week (11-17). Maternal treatment with ACS induces maturation in fetal organs, including the lungs, and reduces the risk of neonatal death by 22% and RDS by 29% in babies born at 24^+0^ to 37^+0^ weeks of gestation (18). However, clinically and preclinically, responsiveness to ACS is variable. Even in infants exposed to the complete course of ACS treatment, some experience little to no benefit and still suffer from severe RDS (19). Despite more than 5 decades of clinical use, there is limited understanding of the pharmacokinetics and pharmacodynamics of commonly used ACS formulations, and the extent to which other factors, including gestational age, fetal sex, fetal growth, and pregnancy complications, impact responsiveness to ACS (18, 20). In part, differences in responsiveness to ACS may be mediated by expression of different isoforms of the GR. Additionally, elevated circulating cortisol concentrations before ACS treatment, which downregulate GR expression, have been associated with poorer responsiveness (21-23). However, the underlying mechanisms driving the variability in ACS response remain poorly defined. This review introduces glucocorticoids and their actions, before discussing the prepartum surge in fetal concentrations of endogenous glucocorticoids and the underlying mechanisms, effects of endogenous and exogenous glucocorticoids on fetal organ maturation, current clinical recommendations for ACS treatment, and potential factors and mechanisms associated with reduced ACS responsiveness in preterm infants.

## Endogenous glucocorticoids

### Introduction

Glucocorticoids are cholesterol-derived steroid hormones that regulate many essential physiological processes in mammals, including glucose metabolism, stress response, immune function, and fetal development (24, 25). Glucocorticoids are synthesized and secreted by the adrenal cortex and exist in the body in multiple forms, which differ between species. Cortisol, also known as hydrocortisone, is the main biologically active endogenous glucocorticoid in humans and sheep, whereas corticosterone is the biologically active endogenous glucocorticoid in rodents (26, 27). Only 5% of glucocorticoids circulate in the unbound, biologically available form, whereas most circulating glucocorticoids are bound to plasma proteins (28). Isoforms of the enzyme 11β-hydroxysteroid dehydrogenase (11β-HSD) can convert glucocorticoids between the active and inactive forms (29). Protein binding in circulation, together with activity of tissue-specific enzymes and the efflux pump P-glycoprotein, regulate glucocorticoid activity by determining their access to GR in the cytoplasm of cells in target tissues (30). Glucocorticoids can bind the GR or the mineralocorticoid receptor (MR) but primarily exert their maturational effects by binding the GR to initiate signaling (31). Interactions of GR with other proteins, such as nuclear factor-κB (NF-κB) and activator protein-1 (AP-1), can also influence cell function (23). Glucocorticoid signaling has a wide array of downstream effects that vary between cell types (32).

### Synthesis and regulation of glucocorticoids

**Figure 1:**
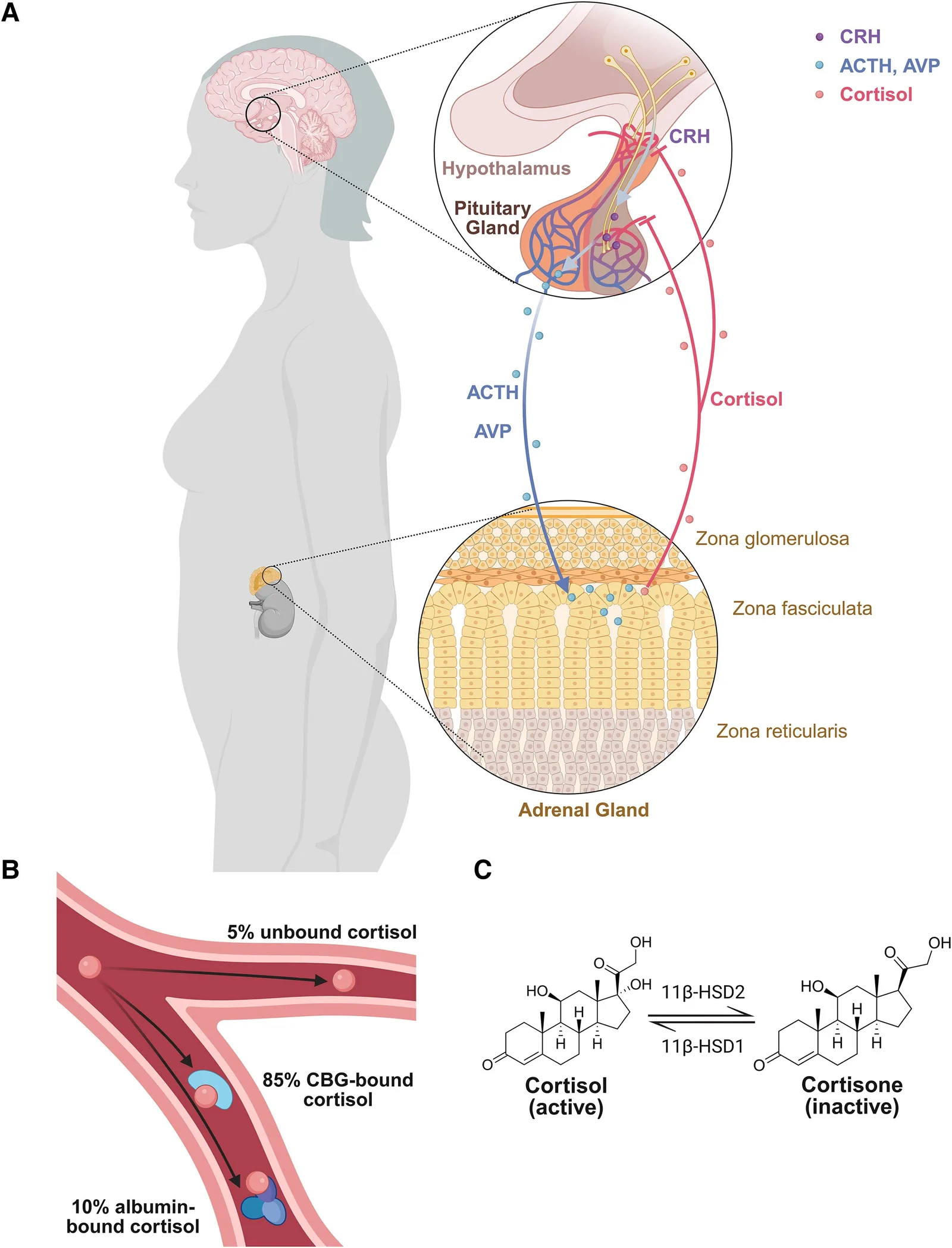
Mechanisms involved in the synthesis, secretion, and sequestration of cortisol in the human body. (A) The HPA axis regulates cortisol synthesis and secretion by the adrenal cortex. The body can produce glucocorticoids in response to physiological or psychological stress. Steroidogenic hypothalamic neurons in the paraventricular nucleus can synthesize and release CRH into the hypophyseal portal vessels to act on the anterior pituitary. The anterior pituitary secretes ACTH and AVP into the circulation, which in turn stimulates synthesis and release of glucocorticoids by the adrenal cortex. (B) Distribution of bound and unbound cortisol in circulation. (C) Interconversion of cortisol (active form) into cortisone (inactive form) by 11β-HSD enzymes.Abbreviations: 11β-HSD, 11β-hydroxysteroid dehydrogenase; ACTH, adrenocorticotropic hormone; AVP, arginine vasopressin; CBG, corticosteroid-binding globulin; CRH, corticotropin-releasing hormone; HPA, hypothalamic-pituitary-adrenal. Created with BioRender.com.

Endogenous glucocorticoids are primarily synthesized and secreted by the zona fasciculata region of the adrenal cortex (Fig. 1A) (33). Glucocorticoids are derived from their precursor, cholesterol, through a series of enzymatic reactions and intermediary steroids. Adrenal secretion of glucocorticoids into the bloodstream occurs in hourly pulses within a circadian pattern. Interactions between the hypothalamic-pituitary-adrenal (HPA) axis, sympathetic adrenal medullary system (SAM), and other hormone circuits mediate the amplitude of glucocorticoid release, with increased amplitude during the active period, that is, day in diurnal animals and night in nocturnal animals (34, 35). The adrenal gland releases pulses of glucocorticoids in response to the pulsatile release of adrenocorticotropic hormone (ACTH) from the anterior pituitary (36). Adrenal secretion of glucocorticoids increases in response to stress, via activation of the HPA axis. Neurons in the paraventricular nucleus of the hypothalamus detect physiological or psychological stressors and secrete corticotropin-releasing hormone (CRH) and arginine vasopressin (AVP) into the hypothalamic-hypophyseal portal circulation (Fig. 1A). These blood vessels carry CRH and AVP to the anterior pituitary where CRH binds and activates corticotropin-releasing hormone receptor 1 (CRHR1) to stimulate the secretion of ACTH by corticotrophs (37). AVP binding vasopressin receptor 1B (V1B) stimulates CRHR1 activation (37). ACTH is released into the systemic bloodstream and acts on the adrenal cortex to stimulate glucocorticoid synthesis and secretion (25). The HPA axis is regulated by negative feedback mechanisms that can downregulate the production and release of CRH and ACTH (23). Glucocorticoids are also synthesized by other tissues, such as the thymus and epithelial barriers, but these locally produced glucocorticoids primarily exert local effects and their regulation is independent of the HPA axis (33, 38, 39).

### Glucocorticoid distribution

Most circulating glucocorticoids are bound to plasma proteins, such as corticosteroid-binding globulin (CBG) and albumin, making them unavailable for binding to the GR (Fig. 1B) (40). Albumin sequesters 5% to 10% of circulating glucocorticoids in the body while 80% to 90% are sequestered through binding to CBG, which has higher glucocorticoid binding affinity than albumin (28, 41). The remaining ~5% of glucocorticoids freely circulate in the bloodstream and are biologically available for activation of the GR. Bound glucocorticoids can be released when proteases such as elastase, produced by neutrophils, cleave these plasma proteins and induce a conformational change that reduces glucocorticoid binding affinity (42). Due to their lipophilic nature, free glucocorticoids readily diffuse through plasma membranes into cells (25).

Glucocorticoid availability to bind GR within the cytoplasm of cells is further regulated by the actions of two 11β-HSD enzymes (Fig. 1C). The first, 11β-HSD1 (type 1) primarily converts inactive glucocorticoids (cortisone in humans) to their active form (cortisol in humans), while 11β-HSD2 (type 2) mediates the reverse reaction (43). To prevent excess binding of biologically active glucocorticoids to MR, which would subsequently disrupt salt and water homeostasis, tissues with high MR expression, such as the kidney and heart, also highly express 11β-HSD2 (29, 44). The MR has 10-fold greater binding affinity for cortisol and corticosterone than GR, so glucocorticoid inactivation by 11β-HSD2 allows MR to be occupied by other hormones under basal conditions (45, 46). Moreover, MR is more selectively expressed in tissues compared to GR, which is expressed in most tissues (31, 47). The lower abundance of MR, and its saturation under basal conditions, means that signaling primarily occurs through GR when there is an increase in glucocorticoid concentrations. Moreover, glucocorticoid transporters such as P-glycoprotein are abundantly expressed in luminal membranes of cell types such as bronchial epithelial cells and capillary endothelial cells in the blood–brain barrier. These transporters can export glucocorticoids to limit activation of downstream pathways (30). Thus, the relative expression of 11β-HSD type 1 and type 2 enzymes, together with expression of efflux pumps that export glucocorticoids, control the availability of glucocorticoids within the cell and consequently GR activation.

### Glucocorticoid receptors

Glucocorticoid receptors (GR) are nuclear ligand-activated transcription factors that regulate expression of glucocorticoid-responsive genes. The human GR is encoded by the nuclear receptor subfamily 3 group C member 1 (*NR3C1*) gene located on chromosome 5 (5q31.3) and contains 9 exons (48). The full-length receptor consists of multiple domains. These comprise a highly variable N-terminal domain (NTD) that contains a ligand-independent activation function-1 (AF-1) region; a hinge domain that contains acetylation motifs; a well-conserved DNA-binding domain (DBD) with 2 C4-type zinc finger motifs, allowing binding to glucocorticoid response elements (GREs); a ligand-binding domain (LBD) that includes a ligand-binding pocket, nuclear localization and receptor dimerization signals; and an AF-2 region that allows interaction with coregulators (23, 25). Differences in domain structure between GR isoforms, such as partial deletions, lead to different functions.

GR isoforms can be generated via alternative splicing of the *NR3C1* transcript or via RNA translation from alternate start sites, with multiple splice and translational isoforms identified in humans, sheep, dogs, guinea pigs, pigs, mice, and rats (reviewed in (23)). Isoform expression and nuclear localization differ between tissues and vary in response to environmental signals (49, 50). Of the identified human transcriptional isoforms (hGRα, hGRβ, hGRγ, hGR-A, and hGR-P), hGRα is the most abundant (51). The hGRα transcript generates the full-length GRα isoform GRα-A (94 kDa), which can induce cell signaling pathways when activated by glucocorticoid binding. Conversely, the GRβ isoform (91 kDa) cannot bind glucocorticoids, because it lacks the 12th α-helix of the LBD due to transcript splicing. Instead, GRβ can act as a dominant negative regulator of GRα signaling pathways by competitively binding coactivators and GREs or by directly binding GRα (52). GRβ is involved in regulating pro-inflammatory genes (52). Both isoforms are expressed in most human cell types and tissues; studies in pregnancy have primarily focused on GRα and GRβ abundance and distribution in tissues such as the placenta (53, 54). The GRγ isoform (95 kDa) exhibits similar regulatory functions as GRα upon glucocorticoid binding but an arginine insertion in the DNA-binding domain reduces both its GRE binding sequence specificity and effect on transcription of target genes (55). The other 2 main GR isoforms are GR-A (65 kDa), where alternative splicing removes exons 5 to 7, and GR-P (70 kDa), where alternative splicing includes part of the intron segment located between exon 7 and 8 (56). GR-A and GR-P each have an incomplete LBD, although how their structural differences impact in vivo function remains unclear. Additional GR transcripts can be generated through alternative translation initiation mechanisms; however, only transcriptional isoforms for GRα have been studied thus far (57). The presence of different GR isoforms is likely to determine the responses of different tissues or individuals to glucocorticoids, although most studies report only GR isoform expression or glucocorticoid sensitivity. There are limited studies linking GR isoform expression with glucocorticoid sensitivity, and most are in the context of diseases such as asthma and rheumatoid arthritis (58-61).

### Glucocorticoid signaling

Glucocorticoids have highly context-specific activity and regulate an expansive repertoire of functions through genomic and nongenomic pathways (32). Binding of glucocorticoids to the GR leads to release of GR-bound proteins such as heat-shock protein (Hsp), Src, and p23 from the GR, enabling the glucocorticoid-GR complex to initiate signaling, predominantly through genomic pathways. The ligand-bound GR can translocate into the nucleus to enhance or repress the expression of target genes by directly binding GREs and/or interacting with other transcription factors (62). Glucocorticoid-bound GRs can regulate gene expression via 3 main mechanisms: direct, tethering, or composite. Firstly, direct binding of the glucocorticoid-GR complex to the GRE induces conformational changes in the chromatin that permit coordinated recruitment of coregulators and chromatin-remodeling complexes. These in turn allow more access by RNA polymerase II to transactivate expression of glucocorticoid target genes (63). The GR can also directly bind negative GREs (nGREs), recruiting corepressors and histone deacetylases (HDACs) to transrepress target gene expression (64). Secondly, through physical interaction with DNA-bound transcription factors such as NF-κB and AP-1, glucocorticoid-bound GR can indirectly tether to the DNA and influence the ability of NF-κB and AP-1 to regulate gene expression. Indirect inhibition of NF-κB and AP-1 is also possible through GR interactions with co-factors such as TIF2/GRIP1, that can repress those transcription factors via largely unknown mechanisms (65), and with enzymes such as O-linked-N-acetylglucosamine transferase, that adds an O-linked-N-acetylglucosamine molecule to the proteins (66). Finally, the glucocorticoid-bound GR can have composite interactions with certain genes and directly bind GRE or nGRE while in association with NF-κB or AP-1 to alter transcription (63).

Activation of the GR by glucocorticoids can also influence cell functions through nongenomic pathways, where the glucocorticoid-bound GR associates with membrane lipids and proteins such as ion channels and glucocorticoid transporters, or with cytoplasmic proteins such as phospholipases and protein kinases (62). Glucocorticoids also have GR-independent effects. For example, glucocorticoids directly interact with membrane lipids and can alter membrane fluidity, impacting ion cycling and ATP use. Glucocorticoid interactions with membrane and cytoplasmic proteins can activate signaling cascades mediated by intracellular Ca^2+^ and kinases such as mitogen-activated protein kinases (MAPKs), hence influencing the regulation of essential cellular processes such as apoptosis, differentiation, and proliferation (67). Furthermore, after the dissociation of GR-sequestering multimeric complexes, some protein components such as Hsp70 and Hsp90 can interact with other cytoplasmic proteins. The implications of these interactions, alongside the ability of the GR to bind to membrane proteins with undefined identities and functions, remain poorly understood (67-70).

## Endogenous glucocorticoids during late gestation

### Introduction

**Figure 2:**
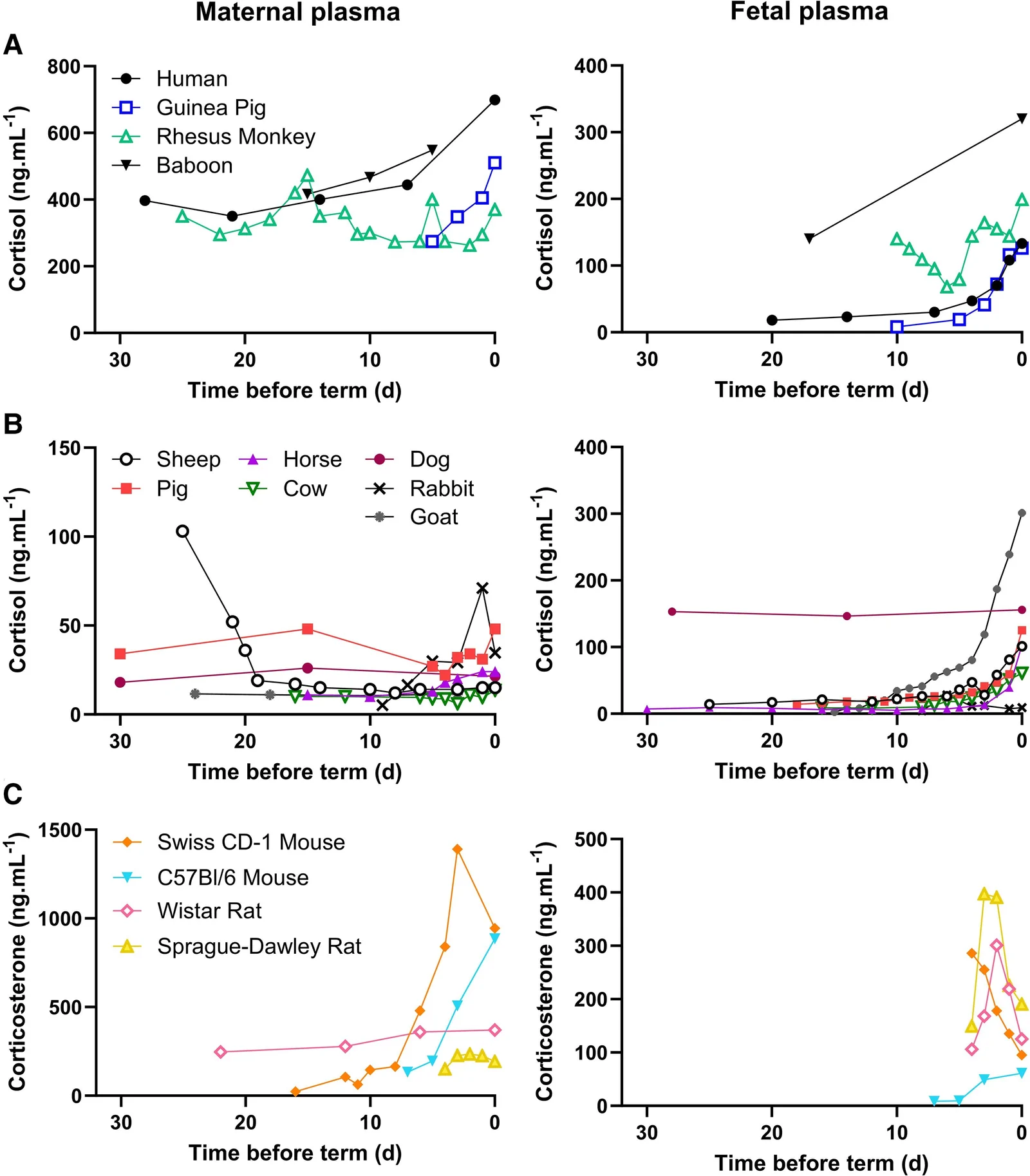
Maternal and fetal plasma concentrations of cortisol and corticosterone in the days leading to term birth in different species. (A) Maternal and fetal concentrations of circulating cortisol in species where fetal circulating cortisol is primarily maternally derived: humans (term 280 gD, 72, 89), guinea pigs (term 63-68 gD, 73), rhesus monkeys (term 165 gD, 74), and baboons (term 185 gD, 75). (B) Maternal and fetal concentrations of circulating cortisol in species where fetal circulating cortisol is primarily fetally derived: sheep (term 140-152 gD, 27), pigs (term 111-120 gD, 76, 77), horses (term 320-360 gD, 78, 79), cows (term 264-296 gD, 80, 81), dogs (term 58-65 gD, 82, 83), rabbits (term 31-33 gD, 84), and goats (term 130-159 gD, 87, 88). (C) Maternal and fetal concentrations of circulating corticosterone in species where fetal circulating corticosterone is primarily fetally derived: mice (Swiss CD-1 and C57Bl/6 strains) (term 19-21 gD, 85, 92) and rats (Wistar and Sprague-Dawley strains) (term 21-23 gD, 90, 91).

Glucocorticoid signaling plays an essential role in fetal development, particularly in late gestation. An increase in circulating glucocorticoid concentrations supports the transition at birth from fetal reliance on placental provision of respiratory, nutritive, and excretory functions to neonatal use of their own physiological systems by promoting maturation of fetal tissues, almost all of which express the GR (71). The prepartum glucocorticoid rise also assists in preparing the mother for parturition in many species. Maternal production of endogenous glucocorticoids is typically tightly regulated, but pregnancy dramatically alters maternal HPA axis activity (24, 32). In most mammalian species, including humans and sheep, there is a surge in circulating fetal glucocorticoid concentrations during late gestation (Fig. 2) (27, 72-92). However, the magnitude and timing of fetal and maternal increases in glucocorticoid concentrations vary between species, and not all species exhibit increases in maternal circulating glucocorticoid concentrations near term (Fig. 2). The mechanisms driving the fetal surge differ between species, with fetal glucocorticoids in late gestation derived from maternal or fetal sources, or a combination of both. The following section describes changes in maternal and fetal glucocorticoid abundance in late pregnancy, and the underlying mechanisms, as well as the important lung maturational effects of glucocorticoid exposure before birth.

### Species with a primarily maternally derived late gestational surge in fetal circulating glucocorticoids

**Figure 3:**
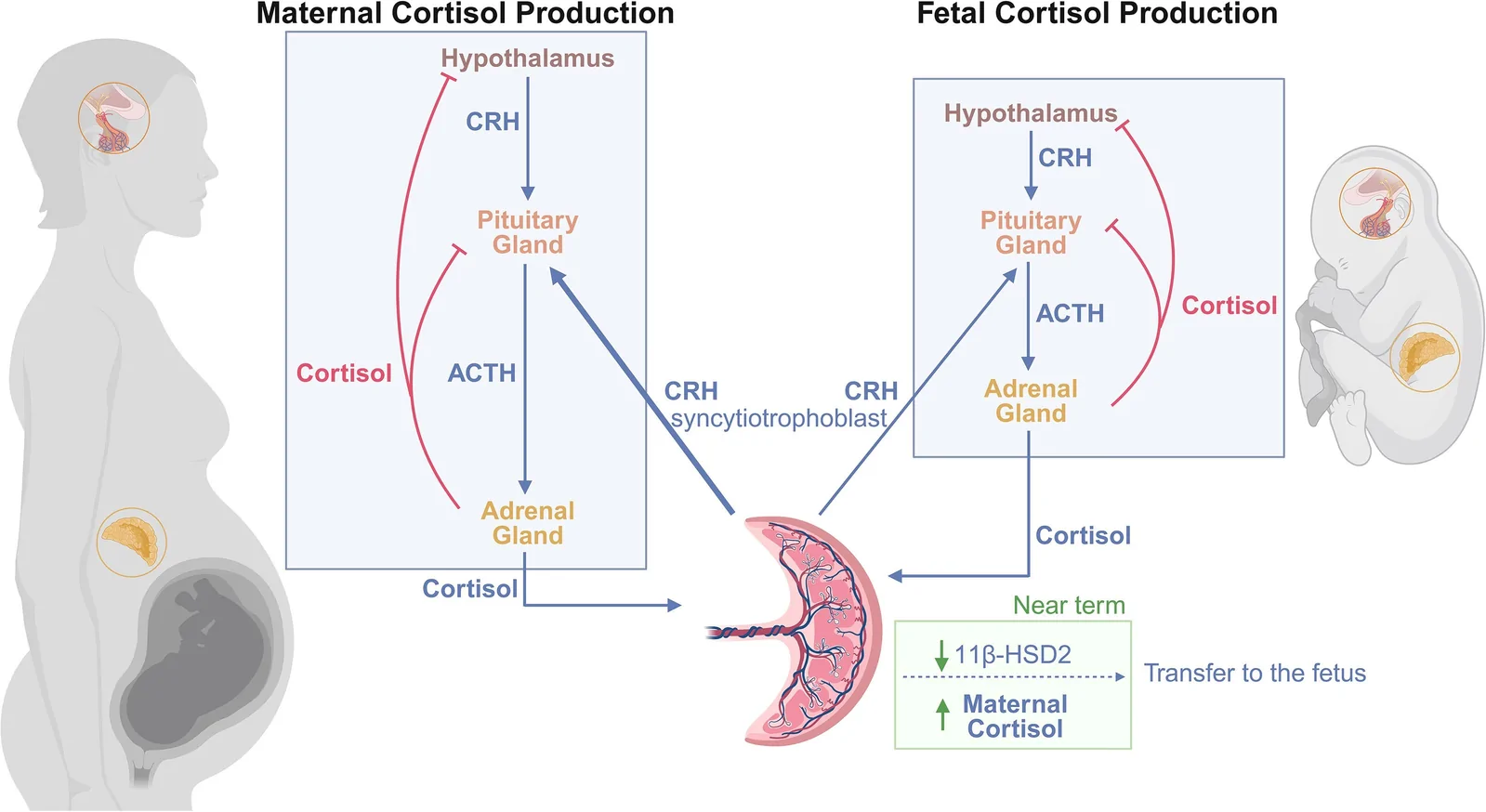
Changes in cortisol regulation by the maternal HPA axis during human pregnancy. Cortisol downregulates production of pituitary CRH but stimulates synthesis and secretion of placental CRH (pCRH). pCRH promotes further cortisol production by the fetus and mother, establishing a positive feedback loop. Maternal cortisol can cross the placenta and enter the fetal compartment, but the fetus is partially protected from early exposure by 11β-HSD2 enzyme activity. Placental 11β-HSD2 activity decreases toward term birth to allow maternal cortisol to reach the fetus, which in conjunction with increasing maternal circulating cortisol, leads to increased cortisol in the fetal circulation. Abbreviations: 11β-HSD, 11β-hydroxysteroid dehydrogenase; ACTH, adrenocorticotropic hormone; AVP, arginine vasopressin; CRH, corticotropin-releasing hormone; HPA, hypothalamic-pituitary-adrenal. Created with BioRender.com.

In humans, the normal negative feedback regulation of circulating CRH by cortisol is overridden in pregnancy due to sequence differences in the promoter region of the CRH gene variants expressed in the hypothalamus and in the placenta. The placental CRH promoter contains retroviral long terminal repeats of the transposon-like human element 1B (THE1B) in the upstream region that act as an enhancer (93, 94). Elevated cortisol downregulates the CRH gene variant expressed in the hypothalamus. In contrast, cortisol upregulates the placental CRH gene variant in syncytiotrophoblasts, specialized epithelial cells that form the barrier between maternal and fetal blood and facilitate nutrient transfer (Fig. 3) (95, 96). Placental syncytiotrophoblasts are the major producer of maternal CRH during the second half of pregnancy (96). This results in a positive feedback loop, allowing simultaneous increases in CRH, ACTH, and cortisol concentrations in the maternal and fetal compartments over the course of gestation (97-99). Placental synthesis and secretion of CRH increases exponentially during the second and third trimester of human pregnancy (100). Placental CRH is mostly secreted into the maternal circulation, replacing the hypothalamus as the primary source of CRH and driving increases in pituitary ACTH production and adrenal gland cortisol production (Fig. 3) (101). To prevent overactivation of the maternal HPA axis, the placenta also produces CRH binding proteins (CRH-BP) that bind CRH and reduce the proportion of free CRH in the maternal circulation to attenuate CRH receptor activation (102). In addition to its effects on maternal cortisol, placentally produced CRH promotes uteroplacental production of estrogen precursors, which are important regulators for several physiological processes involved in preparing the fetus for ex utero life (103, 104) and facilitating parturition (105). As a result of increasing placental CRH production, maternal circulating cortisol increases 2- to 4-fold over the course of normal human gestation and peaks at term, ∼280 days, in humans (Fig. 2A) (72, 106). To avoid early, excessive fetal exposure to maternal cortisol during critical stages of development, the human fetus is protected by high maternal CBG production, which retains cortisol in maternal circulation, and by high placental expression of 11β-HSD2, which converts active cortisol to inactive cortisone, thereby restricting its access and action on the fetus (29). In the last 2 weeks before term birth in humans, between 266 and 280 days of gestation, placental 11β-HSD2 activity declines, which enables maternal cortisol to cross the placenta, resulting in a surge in cortisol concentrations in the fetal circulation (107).

Placental CRH production only occurs in humans and some higher order primates (108-110). In addition to its maternal actions, some placental CRH enters the fetal compartment to increase fetal production of pituitary ACTH and, thus, stimulate fetal adrenal cortisol production (111). The human adrenal cortex begins to form at 4 to 6 weeks after conception (112). Functional specialization of the definitive and fetal zones, which produce cortisol and dehydroepiandrosterone sulfate, respectively, in response to ACTH, occurs at 8 to 10 weeks after conception (112). The fetal adrenal glands rapidly grow from that point and are active from the first trimester, although fetal steroidogenesis is relatively low in early-mid-gestation (113). Activation of the fetal HPA axis at around 28 weeks of gestation increases glucocorticoid synthesis and secretion from the fetal adrenal cortex, acting as a precursor to the prenatal surge, which occurs in the 10 to 15 days before term birth. Together, exposure to cortisol crossing the placenta from the maternal circulation and increasing fetal adrenal cortisol production, result in exposure of the human fetus to increased cortisol concentrations during the last third of gestation, which is important for fetal organ maturation and preparation for delivery.

The increase in fetal plasma cortisol concentrations occurs at similar stages as humans in other primates such as the rhesus monkey (74), baboon (75), chimpanzee, and gorilla (112), which share many physiological and biochemical similarities in prenatal and postnatal development with humans (114, 115). However, there is a dissociation between maternal plasma cortisol and CRH concentrations in some species. Rhesus monkeys have a more modest increase in CRH than humans (109) while CRH concentrations in baboons peak in the first half of gestation and are maintained at high concentrations until after delivery (110). Baboons are unlike other primates as their early CRH increase is accompanied by reduced maternal HPA axis sensitivity to CRH (116) and increases in maternal circulating ACTH and cortisol during pregnancy are, instead, induced by AVP (117). Detailed late gestation data on fetal cortisol concentrations in other primates is limited, with most studies reporting averages in each third of pregnancy. The available evidence shows that fetal circulating cortisol concentrations increase during the third trimester, with surges in fetal circulating cortisol concentrations near parturition in the rhesus monkey and baboon (Fig. 2) (99, 118). Mechanisms contributing to the near term rise in human fetal cortisol, such as increased placental production of CRH and decreased placental 11β-HSD2 expression, also occur in rhesus monkeys and baboons (75, 114). Uteroplacental transfer of maternally produced cortisol also serves as the primary mechanism driving the prepartum rise in fetal cortisol in guinea pigs. Although the guinea pig fetal HPA axis becomes activated during late gestation, fetal adrenal cortisol content does not change (119). In guinea pigs, ∼90% of fetal cortisol is of maternal origin (73, 119, 120), confirmed by assessing transport of radiolabeled cortisol after infusion into the fetal or maternal compartments (121).

### Species with a primarily fetally derived late gestational surge in fetal circulating glucocorticoids

Maternal plasma cortisol concentrations do not rise late in pregnancy in sheep, pigs, cows, dogs, goats and horses; rather, their increases in fetal circulating cortisol concentrations before birth are primarily driven by increasing activity of the fetal HPA axis (97, 122). In all of these species, the adrenal gland forms early in gestation (10). The timing of fetal adrenal activation, when adrenal glands are stimulated to release hormones into the circulation, varies between species, ranging from mid-gestation in sheep to late gestation in pigs to the last few days before birth in horses (27, 122-124). Increased fetal adrenal glucocorticoid synthesis and output are important for fetal organ maturation in these species.

In sheep, pigs, dogs, and goats, the increase in fetal cortisol near term is not driven by placental CRH. Rather, in these species, developmental maturation leads to increased CRH production by the fetal hypothalamus, which, together with increased sensitivity of the fetal pituitary gland to CRH, leads to greater ACTH production, in turn, driving increased cortisol synthesis and secretion by the fetal adrenal gland (76, 125, 126). Additionally, the activity of placental 11β-HSD2 declines near term and allows more maternal cortisol to cross the placenta to the fetal compartment (127-129). In dogs, available data suggest that an increase in fetal cortisol concentration occurs early in gestation at 2 to 4 weeks after conception, with term birth at 58 to 65 days depending on breed (83), although the literature is limited to studies where cortisol was measured at fortnightly or less frequent intervals (79, 130). Horses have fluctuating maternal cortisol concentrations throughout pregnancy and their fetuses have little to no change in circulating ACTH, cortisol, or CBG capacity across most of gestation (79, 130). The fetal foal's adrenal cortex is insensitive to ACTH until around 5 days before term birth, when there is a sharp rise in ACTH and a subsequent surge in circulating fetal cortisol that induces rapid fetal maturation (130, 131). This, alongside a decline in plasma CBG in the mother, contributes to the sudden increase in concentrations of free cortisol in the equine fetal circulation (130).

The fetal adrenal is also the main source of glucocorticoids in the fetus of most rodents, including mice and rats. The adrenal glands of mice and rats lack the cytochrome P450 family 17 (CYP17) enzyme that catalyzes the conversion of cholesterol and pregnenolone precursors to cortisol, and corticosterone is the active steroid in these rodents (132, 133). The regulation of fetal corticosterone concentrations in mice and rats is primarily driven by fetal HPA axis activity, although maternal circulating corticosterone concentrations do increase throughout pregnancy. Circulating corticosterone concentrations in the pregnant mouse begin to increase during the second half of pregnancy and a sharp surge occurs around 5 days before parturition (133), increasing to over 50-fold higher than nonpregnant adults (134). Maternal plasma corticosterone concentrations also increase during late pregnancy in the rat, with a sharp rise around mid-gestation followed by a steady increase occurring in the few days leading to term birth at 21 days gestation (135). However, unlike most other species with increasing circulating maternal glucocorticoid concentrations over gestation, the direction of placental glucocorticoid transfer in the rat is primarily from the fetus to the mother, not the mother to the fetus (85, 133, 135-137). In addition, the extent of glucocorticoid transfer and increase varies depending on the mice or rat strain (85, 90-92). Although circulating corticosterone concentrations in the maternal rat double by mid-pregnancy, the Wistar strain has higher maternal, but lower fetal, corticosterone concentrations than the Sprague-Dawley strain (90, 91). In both mice and rats, the fetal HPA axis becomes active around 5 days before term birth, although changes in fetal corticosterone concentrations differ between these species (Fig. 2C) (138). Interestingly, fetal exposure to elevated glucocorticoids occurs at much earlier stages of organ maturation in these rodents compared to many other mammals. For example, human lungs enter the alveolar stage of development before term birth (139) while mice are born with lungs at the saccular stage of development, with alveoli development and maturation occurring postnatally (140). Thus, endogenous glucocorticoid exposure occurs at earlier stages of organ maturation in rodents than in other species due to differences in the timing of organ maturation relative to birth. In early gestation, high 11β-HSD2 expression in the mouse and rat placenta inhibits uteroplacental transfer of corticosterone from fetal to maternal circulations (135, 138). Nearing term, the expression of 11β-HSD1 in the placental syncytiotrophoblast increases, whereas expression of 11β-HSD2 decreases, allowing fetally produced corticosterone to enter the maternal circulation and contribute to the initiation of parturition (141, 142). The transfer contributes to a significant portion of corticosterone in the maternal circulation, with Barlow et al reporting that ∼20% of maternal corticosterone in the rat was of fetoplacental origin (133). Adrenalectomy of pregnant rats did not modify maternal nor fetal serum corticosterone or CBG concentrations (143), suggesting that the rat fetus produces sufficient amounts of corticosterone to induce maturational effects without maternal contributions (135). Most studies report decreases in circulating corticosterone concentrations in the last few days of gestation in the fetal mouse and rat. This likely reflects greater negative feedback of corticosterone at the fetal hypothalamus, due to increasing hypothalamo-hypophyseal sensitivity to glucocorticoids, alongside continued corticosterone transfer to the mother (138, 144, 145). Low circulating corticosterone concentrations are maintained well into the postnatal period in these rodents (146).

### Maturational effects of the prepartum glucocorticoid surge

#### Introduction

The prenatal rise in fetal circulating glucocorticoids is critical for preparing the fetus for extrauterine life. During mid-gestation in humans, the expression of GR increases in essential fetal organs such as the lung, heart, gut, and brain (23). Glucocorticoid activation of developmental signaling pathways, mediated by GR, promotes the switch from cell proliferation to differentiation and matures the fetal respiratory, cardiovascular, and gastrointestinal systems, in addition to promoting closure of the blood–brain barrier.

#### Respiratory system

**Figure 4:**
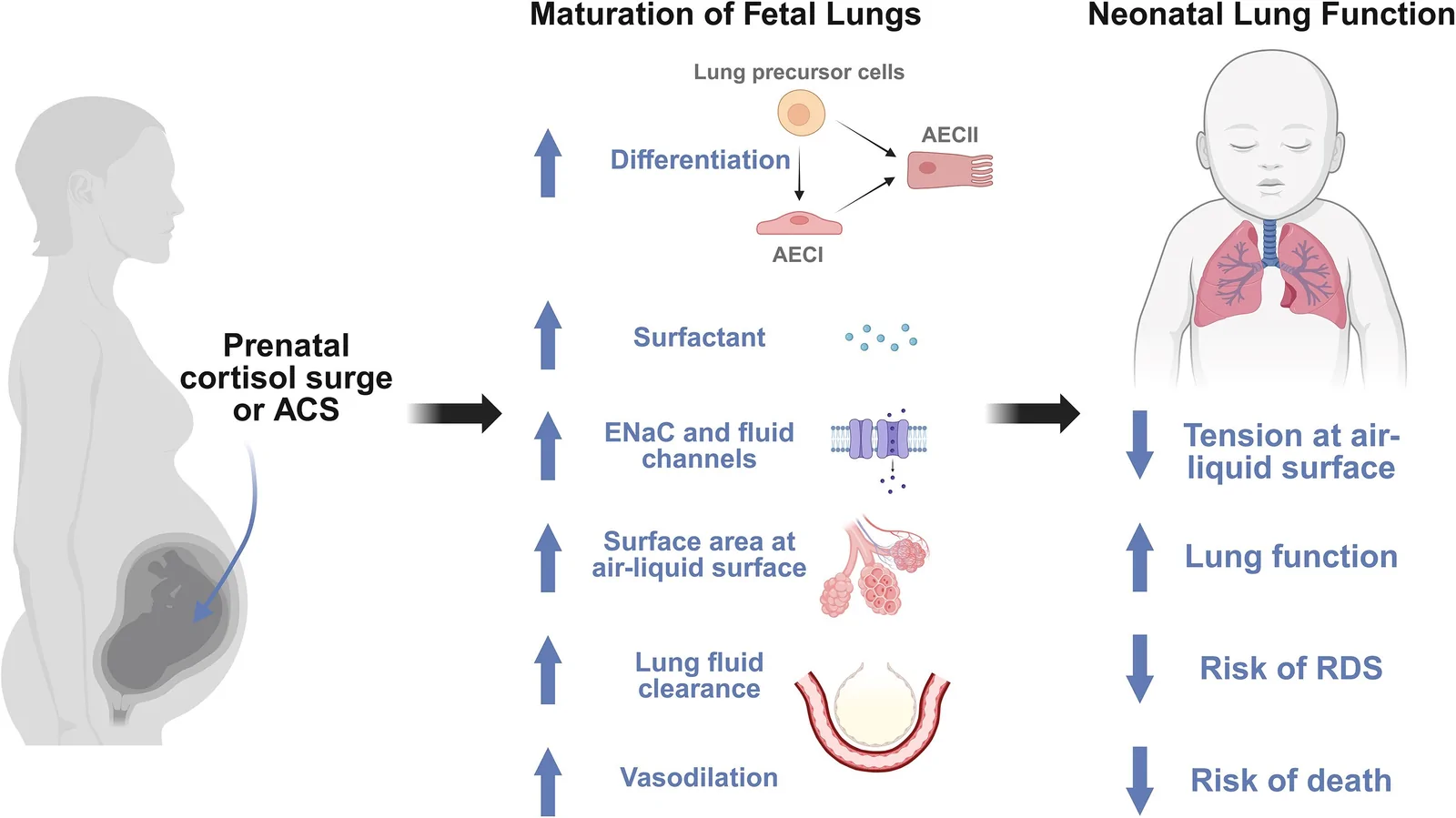
The prenatal surge in fetal plasma glucocorticoid concentrations triggers lung maturational processes that improve lung function after birth. Abbreviations: ACS, antenatal corticosteroids; AECI, alveolar type I epithelial cells; AECII, alveolar type II epithelial cells; RDS, respiratory distress syndrome. Created with BioRender.com.

Glucocorticoid-induced maturation of the fetal lung prepares the fetus for the transition from placental to pulmonary gas exchange (147, 148). Glucocorticoid-GR signaling induces pathways that lead to thinning of the air-blood diffusion distance, clearance of fetal lung fluid and production of surfactants to lower tension at the air-liquid surface, enabling efficient gas exchange in the newborn lung (Fig. 4) (71). Glucocorticoids stimulate the differentiation of lung precursor cells into type I alveolar epithelial cells (AECI), which are large, extremely thin cells that establish a large surface area for gas exchange, and type II alveolar epithelial cells (AECII), which are small cuboidal cells that maintain the alveolar surface by producing pulmonary surfactant, cytokines, and chemokines (149-153). AECI cover ∼95% of the alveolar surface, minimizing the distance between the alveolar airspace and blood by forming a thin alveolar-capillary membrane that permits efficient gas exchange (154). While AECII comprise only around 5% of the alveolar surface in mammals, they account for around 60% of the total number of AEC in the lung (151). AECII synthesize, secrete, and recycle pulmonary surfactant components (surfactant proteins, phospholipids, and neutral lipids), lowering air-liquid tension at the gas exchange surface and, subsequently, preventing alveolar collapse (155). The prenatal surge in glucocorticoids stimulates several lung maturational effects, including strongly promoting AECII differentiation and upregulation of surfactant protein expression (156, 157). In addition to initial differentiation from lung precursor cells, the AEC phenotype has plasticity. Glucocorticoids play a role in regulating transdifferentiation of AECI to AECII, and vice versa, in response to physical strain (158). Expansion of the fetal lung stimulates differentiation of AECII to AECI, whereas decreased expansion can promote differentiation of AECI to AECII (159-161). As a result, the relative proportion of AEC phenotypes in the lung epithelium changes across gestation, with shifts in the promotion of AECI or AECII differentiation occurring at defined stages of fetal lung development. In fetal sheep, AECI differentiation is favored during the late canalicular stage of lung development, when the respiratory bronchioles form and vascularize. There is a shift to AECII differentiation once there is a surge in glucocorticoids, which also promotes alveoli maturation and surfactant production to support gas exchange (156). Glucocorticoid signaling also regulates the differentiation of other cell types, such as pulmonary mesenchymal cells, and their activity (162).

Throughout gestation, the fetal lungs are filled with fluid to maintain the lungs in an expanded state and stimulate lung growth (163, 164). This lung fluid must be cleared at birth to allow pulmonary gas exchange. Transport of lung fluid from the alveolar space to the interstitial space assists in maintaining alveolar stability and preventing flooding of interstitial fluid into the airspace (71). The process of fetal lung fluid clearance is stimulated by the rise in glucocorticoids during late gestation (147, 148). Fetal fluid resorption occurs through active transepithelial ion transport, primarily via epithelial sodium channels (ENaC), a critical sodium transporter expressed on the surface of AEC that establishes an osmotic gradient to move water from the airspace to the interstitial spaces (165, 166). Fluid is then cleared from the interstitium into the vasculature via water movement by aquaporins (AQP) expressed on the epithelium and vascular endothelium (167, 168). Glucocorticoids increase ENaC expression in the apical membrane of AECI and AECII (166), AQP5 expression in the apical membrane of AECI, and AQP1 expression in the alveolar capillaries (167).

Consistent with the importance of glucocorticoids for these processes, preventing the prepartum surge in fetal circulating glucocorticoids inhibits lung development (169-171). Bilateral fetal adrenalectomy in sheep fetuses prevents the rise in fetal plasma cortisol concentrations from about 7 days before birth, despite increases in circulating ACTH (169). These adrenalectomized sheep fetuses have impaired fetal lung liquid secretion and resorption (171), fewer AECII, and poor lung function (170) after preterm delivery, compared to intact fetuses. Consistent with the important role of GR activation in the fetal lung for lung maturation, 90% of newborn GR-knockout mice die at birth due to atelectatic lungs, leading to respiratory failure (172). Because human preterm birth occurs before the prepartum surge in endogenous corticosteroids, preterm infants have immature lungs that are unprepared for independent, ex utero respiration.

#### Cardiovascular system

Glucocorticoids enhance maturation of the cardiovascular system by acting on GR in the heart to promote maturation, as well as on blood vessels where vasoconstriction leads to increased blood pressure (173, 174). Cardiomyocytes, specialized heart muscle cells responsible for generating contractile force, undergo rapid proliferation during early fetal development to increase the number of heart muscle cells (174). During late gestation in sheep and humans, the fetal glucocorticoid surge promotes production of thyroid hormone, and these 2 hormones induce a transition in cardiomyocyte phenotype from proliferative to hypertrophic growth (175-177). Thus, humans and sheep have increasing cardiomyocyte endowment throughout gestation (178, 179), but cardiomyocyte endowment becomes fixed at birth due to the limited capacity for further cardiomyocyte proliferation (174, 180). The normal enlargement of cardiomyocytes supports the increased functional load on the heart after birth (181), but if cardiomyocyte endowment is reduced, these cells may become larger at earlier stages in postnatal life, resulting in a reduced ability to respond to increases in cardiac load later in life (182). The glucocorticoid surge also induces expression of oxidative phosphorylation machinery in cardiomyocytes in late gestation sheep, supporting the switch from glycolytic metabolism to fatty acid oxidation to meet their high postnatal energy requirements (177, 183-185). The fetal glucocorticoid rise also promotes the maturation of the complex cardiac conduction system, which allows the atrial and ventricular chambers of the heart to propagate electrical activity and coordinate synchronous rhythmic contractions to pump blood throughout the circulation (186).

The prenatal glucocorticoid surge is also important for regulation of blood pressure (173, 187). The fetal glucocorticoid surge elevates the arterial setpoint, the central regulator of resting arterial blood pressure, by increasing baroreflex sensitivity (188), vascular responsiveness to the renin-angiotensin-aldosterone system (189, 190), and cardiac and smooth muscle contractility (191). Glucocorticoids also facilitate increases in femoral and peripheral vascular resistance, which is essential to prepare the vasculature to maintain arterial pressure when the placental circuit is removed (173, 192). Thus, without exposure to the prepartum glucocorticoid surge, infants who were born preterm can have immature hearts with fewer and larger cardiomyocytes, impaired adaptability to stressors, lower diastolic function after birth, and poor contractility (193-196).

#### Gastrointestinal tract

The prenatal glucocorticoid surge triggers developmental changes in the fetal gastrointestinal (GI) tract in anticipation of the shift from placental nutrition to enteral nutrition after birth (197). Glucocorticoids promote the formation of tight junctions in the small intestine to increase the integrity of the intestinal barrier and inhibit pathogen invasion (198, 199). The capacity of the small intestine for nutrient uptake depends in part on its surface area, which is much larger than the cylindrical surface due to the presence of villi and microvilli on its surface. Glucocorticoids influence villus-crypt architecture in the small intestine, increasing villus height and crypt depth to increase its surface area (200). Nutrient absorptive capacity is further enhanced by glucocorticoids through stimulating enterocyte expression of nutrient transporters required for facilitated transport, such as glucose transporter protein 2 (GLUT2) and GLUT5 (201-203), or active transport, such as amino acid transporters and sodium-glucose cotransporter 1 (SGLT1, 203, 204). These transporters allow the enterocytes lining the GI tract to uptake nutrients from the lumen and release them into the bloodstream. The fetal surge also promotes digestive capacity of the GI tract (205, 206). Glucocorticoids promote the secretion of stomach acid and gastrin, which activate gastric proteolytic enzyme activity in the postnatal period and also assist in preventing bacterial colonization of the upper GI tract (205). The fetal surge in glucocorticoids also leads to increased activity of digestive enzymes such as chymosin and pepsin in the stomach, amylase in the pancreas, and lactase and aminopeptidase in the small intestine (197, 205). Thus, preterm infants who miss the fetal glucocorticoid surge have reduced absorptive capacities and limited intestinal motility compared to term infants (207). Necrotizing enterocolitis, characterized by ulcerative inflammation of the intestinal wall, is usually associated with an underdeveloped gut and is one of the most common conditions seen in preterm infants (208, 209). Reflecting the importance of glucocorticoids for gastrointestinal maturation, the risk of necrotizing enterocolitis in preterm babies was halved in those exposed to ACS, compared to untreated babies (18).

#### Brain

Brain development begins in the embryonic period, with growth continuing postnatally and maturation extending into adulthood (210-212). Glucocorticoids can diffuse through the blood–brain barrier's network of specialized cerebrovascular endothelial cells and neighboring astrocytes, neurons, and pericytes to target GR (213, 214). Glucocorticoids have region-dependent effects within the brain, because the GR is differentially expressed throughout the brain, with high expression in the amygdala, hippocampus, and prefrontal cortex (215). The blood–brain barrier is a highly-selective semipermeable interface that regulates the transfer of molecules between the circulation and brain and prevents some harmful substances from entering the brain (216). The prenatal glucocorticoid surge promotes maturation of this barrier by decreasing membrane permeability (217). Glucocorticoid exposure in late gestation enhances expression of tight junction proteins such as claudin-1, occluding, and zonula occludens 1 (ZO-1) in cerebral endothelial cells to reduce paracellular permeability (218). Glucocorticoid-induced upregulation of the efflux transporter P-glycoprotein (219, 220), which is expressed at the blood–brain barrier, reduces uptake of corticosteroids such as cortisol and dexamethasone into the brain, although interestingly not corticosterone (221). The prenatal glucocorticoid surge also stimulates maturation of the microvasculature to support blood–brain barrier integrity, particularly within the germinal matrix, a temporary region that is a highly vascularized site for glial and neuronal differentiation (222, 223). In humans, the germinal matrix disappears at 36 weeks of gestation (224). Intraventricular hemorrhage (IVH) is a common major neurologic complication associated with preterm birth, particularly preterm infants born before 32 weeks of gestation (225). In preterm infants, the hemorrhage originates in the germinal matrix's fragile capillary network and bleeds into the ventricles (225). The use of ACS lowers the risk of IVH in preterm infants, likely due to improved blood–brain barrier function; however, the direct mechanisms still require further study (226-228).

## Antenatal corticosteroids

### Introduction

The immaturity of physiological systems contributes to the greater risk of short-term morbidity and mortality in preterm infants born before the fetal surge in glucocorticoids. The administration of ACS reduces morbidity and mortality in preterm infants by inducing similar maturational effects as the prepartum surge in endogenous glucocorticoids.

### Preterm birth

Globally, 1 in 10 babies, an estimated 13.4 million annually, are born preterm. Rates of preterm birth have barely changed within the past decade, ranging from 4% to 16% depending on country (2). The World Health Organization (WHO) classifies preterm babies based on gestational age at birth: extremely preterm when born at <28 weeks completed gestation, very preterm at 28 to <32 weeks, moderate preterm at 32 to <34 weeks, and late preterm at 34 to <37 weeks (229). Approximately 85% of preterm infants are born during the moderate to late preterm period (4). Interestingly, males are more likely to be delivered preterm than females (230). Although the incidence and severity of short-term morbidities is lower in moderate to late preterm infants compared to earlier preterm infants, late preterm infants are still at greater risk than full-term infants (231). Complications due to preterm birth are the leading contributor to morbidity and mortality among children under 5 years of age (229). In a study conducted in Canada from 1983 to 1996, a period mostly before the widespread adoption of ACS, the mortality rate in the first year after birth was 2.6% for preterm infants (26.2% for extremely preterm, 6.0% for very preterm, 2.4% for moderate preterm, and 0.9% for late preterm), compared to 0.2% for infants born at term (232).

One of the main organs important for surviving the transition to ex utero life is the lung. Babies born before 36 weeks of gestation have surfactant-deficient lungs, resulting in increased alveolar surface tension and risk of alveolar collapse (atelectasis), while babies born before 28 weeks of gestation lack alveoli, instead relying on alveolar ducts and buds that are very inefficient at gas exchange (139). These lung immaturities mean that severe respiratory morbidities, such as the respiratory condition RDS, are more common in preterm infants (18). Early diagnosis of RDS is crucial, although diagnostic criteria varies greatly due to the lack of a consensus definition of RDS. Clinical signs and symptoms associated with RDS include, but are not limited to, tachypnea, expiratory grunting, nasal flaring, and subcostal and sternal retractions commencing shortly after birth and increasing in severity across the first 48 hours of postnatal life (233, 234). RDS is a result of lung immaturity, mostly caused by insufficient surfactant at birth, that increases the risk of alveolar collapse on exhalation and thus increases the amount of inspiratory effort required to inflate the newborn's lungs (157). Increased surface tension increases the amount of pressure required to maintain alveolar shape and, eventually, there is damage to the respiratory epithelium (71). Without ACS treatment, ∼45% of preterm infants born between 23 and 33 weeks develop RDS (9). The incidence of RDS is inversely proportional to gestational age, with RDS occurring in ∼60% of babies born between 26 and 28 weeks of gestation, declining to ∼20% in babies born between 34 and 36 weeks of gestation (235, 236). Preterm males consistently have worse short-term and long-term pulmonary outcomes than their female counterparts, with males having approximately 1.7-fold higher RDS incidence than females (237, 238). Even with advanced neonatal care, preterm infants born before 34 weeks of gestation without ACS remain at high risk of RDS and other common morbidities of preterm birth, including chronic conditions such as bronchopulmonary dysplasia, which occurs in over 30% of infants born before 30 weeks of gestation (7). Bronchopulmonary dysplasia in contemporary preterm cohorts reflects impaired lung development, resulting in an immature lung phenotype with fewer and simpler alveoli, less fibrosis and distribution of damage throughout tissues (239).

Preterm birth continues to adversely impact overall health even after the neonatal period. A systematic review by Diggikar et al reported that preterm infants have significantly higher risk of developing respiratory infections in the first 2 years after birth compared to infants born at term (relative risk [RR]: 1.52 [95% CI 1.25-1.85] (240)). The risk of adverse outcomes increases proportionally with degree of prematurity, and the impacts of preterm birth can continue through childhood and adulthood (241). Preterm birth also impacts nonrespiratory health outcomes. A systematic review of neurodevelopmental outcomes by Allotey et al (6) reported that measures of cognitive performance in primary and secondary school-aged children born preterm were consistently lower than those of children born at term. Compared to term-born children, children born preterm had poorer working memory (primary school-aged: standardized mean difference [SMD] −0.61 [95% CI −0.72 to −0.50]; secondary school-aged: SMD −0.53 [−0.72 to −0.34]), processing speed (primary school-aged: SMD −0.53 [−0.66 to −0.41]; secondary school-aged: SMD −0.35 [−0.58 to −0.13]), and motor skills (primary school-aged: SMD −0.59 [−0.89 to −0.28]; secondary school-aged: SMD −0.44 [−0.50 to −0.37]). Moreover, prematurity was associated with greater likelihood of diagnosis of developmental disorders such as attention-deficient/hyperactivity disorder (ADHD, odds ratio: 1.6 [1.3-1.8]), with a 3-fold higher rate in very and moderately preterm-born children compared to those born at term (6). Most adults who were born preterm are generally healthy although there is evidence that neurodevelopmental impacts of preterm birth persist (242). Some studies report that adults born at different stages of prematurity have similar cognitive performance to their full-term counterparts (243, 244) or have minor defects such as weaker working memory (245, 246). Adults born preterm have abnormalities in brain structure and reduced white and gray matter volumes in multiple brain regions, including the temporal, frontal, insular, and occipital areas (247-250). Some studies show correlations between physical abnormalities and functional outcomes, suggesting that prematurity has life-long impacts on the brain (242), but they are inconsistent and highlight the need for further study.

### Effects of antenatal corticosteroids on preterm lung development

While ACS acts on multiple organs, the fetal lungs are a critical target. Human lungs form early in development, at around 4 weeks after conception, and begin to express GR mRNA from as early as 10 weeks after conception (ie, from 12 weeks gestation, 251). In infants born preterm, ACS are administered to promote organ maturation, with similar effect to the rise in endogenous glucocorticoids that occurs near term birth. ACS improves respiratory function through multiple mechanisms, one being the promotion of surfactant production. ACS treatment increases surfactant synthesis in human lung epithelial cell cultures; upregulating mRNA expression of SP-A (252), SP-B (253), and SP-C (254) compared to untreated controls. In human fetal lung explants, SP-B and SP-C mRNA abundance were approximately 4- and 30-fold higher, respectively, in ACS-treated explants compared to controls (255).

Defining the impact of ACS itself on outcomes for preterm babies in the neonatal intensive care unit (NICU) is complex due to the use of multiple interventions, which have changed over time. Although in vitro studies allow short-term responses to be assessed, they do not allow functional and molecular mechanisms to be assessed concurrently, necessitating use of preclinical in vivo models. Use of preclinical in vitro models also overcomes limited access and availability of human fetal tissues. Treatment of moderately preterm sheep with the current consensus clinical dosing regimen used in Australia and New Zealand (maternal intramuscular administration of a betamethasone phosphate-acetate mixture at 48 and 24 hours before delivery, 17), results in higher mRNA expression of surfactant proteins A, B, and C and of the fluid transporter aquaporin 5, compared to untreated fetuses (256). Treatment with ACS also induces structural changes in the preterm sheep lung, including thinning of alveolar walls, lower alveolar wall volume fraction, and higher proportional area of alveolar ducts, which translate to better dynamic and static lung compliance, and enhanced gas exchange (257-259). In sheep, comparisons of continuously infused or maternally injected ACS preparations have been used to demonstrate that the lung maturational effects of ACS depend on duration of fetal exposure to elevated steroid concentrations (258). Seven days after treatment commenced, preterm lambs exposed to 2 maternal intramuscular doses of 1:1 betamethasone phosphate + betamethasone acetate mix, given 24 hours apart, had better lung compliance than controls, whereas those who received only a single treatment did not benefit (258). Alongside increased surfactant production, ACS treatment accelerates AECII maturation and increases AECII proportion in cultured populations of human AEC (149) and lung tissue of preterm rabbits (150). In the human lung, ACS treatment promotes alveolarization by accelerating the normal thinning of the double capillary loops that form the gas exchange surface of the alveoli (260). However, ACS-treated preterm rabbits have lower total alveoli number than untreated controls because there is a cessation of alveolarization after the initial response, with inhibition of secondary septa outgrowth (261). The expression of multiple subunits of ENaC and Na^+^-K^+^-ATPase transporters involved in sodium transport in epithelial cells that facilitate fluid resorption in the lung, is also stimulated by ACS in isolated rat lung cells (262). Preterm fetal sheep with ACS infused 48 hours before delivery also had higher ENaC mRNA expression than untreated fetuses (263). These molecular changes contribute to improved neonatal lung function after ACS treatment (150, 264).

There are concerns that current clinical ACS regimens exceed the minimum effective ACS dose for inducing maturation in the fetal lung (265). Recent animal studies demonstrate that improved lung maturation requires prolonged ACS exposure, and lower betamethasone doses than used clinically are sufficient to induce similar lung maturational benefits (264, 266, 267). Additional studies are required to assess outcomes in other organs.

### Types and formulations of antenatal corticosteroids

The therapeutic efficacy of ACS depends on drug activation, efficiency of maternal-fetal transfer and systemic clearance rate. Betamethasone (9α-fluoro-16β-methyl-1,4-pregnadien-11β,17,21-triol-3,20-dione) and dexamethasone (9α-fluoro-16α-methyl-1,4-pregnadien-11α,17,21-triol-3,20-dione) are potent synthetic glucocorticoids. These compounds are stereoisomers, sharing the same chemical formula but differing in the spatial orientation of a single methyl group, resulting in differing GR affinities and clearance rates. Betamethasone and dexamethasone have, respectively, 5.4- and 7.1-fold higher affinity for GR than cortisol, allowing for stronger and extended GR activation that supports sustained expression of factors involved in fetal lung maturation (Table 1) (268-275). Moreover, unlike cortisol, betamethasone and dexamethasone lack affinity for MR, minimizing activation of off-target pathways, and for CBG, which increases their bioavailability (276). These synthetic glucocorticoids are also minimally oxidized by 11β-HSD2 in the placenta due to the 9α-fluoro and 16α-methyl/16β-methyl substitutions, allowing rapid placental transfer and fetal exposure (277). In an ex vivo human placental perfusion study, ∼16% of betamethasone and ∼14% of dexamethasone that entered the maternal circulation reached the fetal compartment over a 3-hour period (278). As a result of these pharmacological properties, maternally administered betamethasone and dexamethasone activate the fetal GR to a much greater extent than cortisol.

**Table 1:** Pharmacokinetics of synthetic glucocorticoids in humans

Steroid	Relative GR affinity (268)	Half-life (T_1/2_) in maternal circulation	Fetal/neonatal exposure after maternal treatment
Cortisol	1	Nonpregnant: T_1/2_ = 69.5 minutes (269) Pregnant: T_1/2_ = 104.6 minutes (269)	Data not available
Dexamethasone phosphate	7.1-fold	Nonpregnant: After single dose of 6 mg (i.m.), T_1/2_ = 5.2 ± 0.4 hours (270) After single dose of 6 mg (oral), T_1/2_ = 5.5 ± 1.2 hours (270) Pregnant: After 2 doses of 8 mg (∼24 hours interval, maternal i.m.), T_1/2_ = 2.37 hours (271)	After single dose of 8 mg (maternal oral), dexamethasone concentrations in umbilical artery averaged 26 μg/L after 8-11 hour (272) After four 5 mg doses (∼12 hour intervals, maternal i.m.), dexamethasone concentrations in umbilical artery averaged 6.5 ng/mL in babies delivered <12 hours after the last dose. Dexamethasone was undetectable in neonates at 24 hours post-delivery (273)
Betamethasone phosphate	5.4-fold	Nonpregnant: After single dose of 6 mg (i.m.), T_1/2_ = 10.2 ± 2.5 hours (270) After single dose of 6 mg (oral), T_1/2_ = 13.9 ± 7.5 hours (270) Pregnant: Data not available	Data not available
Betamethasone phosphate + betamethasone acetate (1:1)	5.4-fold	Nonpregnant: After single dose of 6 mg (maternal i.m.), T_1/2_ = 59 ± 35 hours (270) Pregnant: After single dose of 12 mg (maternal i.m.), T_1/2_ = 6 hours (274)	After 2 doses of 12 mg (∼24 hours interval, maternal i.m.), undetectable in cord blood by 62-72 hours after first dose (274) After 1-3 doses of 12 mg (∼24 hours interval, maternal i.m.), betamethasone concentrations in umbilical artery (3.3-8.0 ng/mL) were similar to those in maternal peripheral blood (275)

Abbreviations: GR, glucocorticoid receptor; i.m., intramuscular; T_1/2_, half-life.

Different formulations of ACS and routes of delivery result in different fetal exposure patterns (Table 1) (265). Betamethasone is clinically used in 2 main formulations: as a rapid-release phosphate ester prodrug or a combined phosphate-acetate suspension that allows both immediate and sustained drug release. The addition of phosphate makes betamethasone more water-soluble, allowing an intense but short peak in bioavailability and GR activation, whereas the addition of acetate leads to limited solubility that delays entry and subsequent hydrolysis, allowing prolonged GR activation in tissues (265). A 1:1 betamethasone phosphate to betamethasone acetate mix was used in the first clinical trial and has remained in widespread clinical use (18, 279). Dexamethasone is usually given as a phosphate ester alone, without a slow-release component (280). Dexamethasone phosphate is therefore given as 4 doses at 12-hour intervals to maintain GR activation concentrations over a 48-hour period (268). A sodium phosphate salt form of betamethasone and dexamethasone may be used in formulations to increase water solubility, with similar activity as the phosphate ester forms (18). Women receiving oral dosing achieve peak circulating ACS concentrations more rapidly than those receiving intramuscular administration, although route of administration does not significantly impact half-life (270).

The differences in formulation also impact clearance. When delivered via maternal intramuscular injection, betamethasone phosphate has a terminal half-life of 11 hours, nearly double the duration of dexamethasone phosphate, which has a half-life of 5.5 hours, but substantially lower than the 1:1 betamethasone phosphate + betamethasone acetate combination, which has a half-life of 60 hours (Table 1) (270). ACS is rapidly cleared from the fetal compartment by P-glycoprotein, unlike the endogenous glucocorticoid surge that occurs naturally in late gestation (281). However, the pharmacokinetics and pharmacodynamics of ACS require further study, particularly in the fetus, despite the widespread adoption of ACS several decades ago. The human data is largely based on older studies with limited assay sensitivity and few timepoints and sample numbers for fetal steroid determination, which are limited to cord blood samples collected at delivery. It is interesting to note that pharmacokinetics differs between singleton and twin pregnancies, with evidence of a shorter half-life and greater clearance in twins (282). Further study is needed to understand the maturational benefits of customizing ACS dose for multiple gestations.

### Translation of antenatal corticosteroids to clinical practice

#### Preclinical discovery and first clinical trial

Due to their anti-inflammatory properties, synthetic glucocorticoids are widely used in clinical settings to manage conditions including chronic diseases such as asthma, osteoporosis, and rheumatoid arthritis (283-285). The capacity of glucocorticoids to induce maturation of the fetal lung was a serendipitous discovery. In the 1960s, Dr. (later Professor Sir) Graham (Mont) Liggins was studying the role of glucocorticoids in the initiation of labor in sheep. He observed that when pregnant sheep were treated with glucocorticoids, preterm birth was induced but that some lambs were delivered with lungs capable of inflation, allowing survival at pre-viable gestational ages (286). At that time, there was a 50% mortality rate in infants diagnosed with RDS and the first mechanical ventilators to provide infant breathing support were being introduced (287). Hypothesizing that these lung maturational effects of exogenous glucocorticoids in sheep may also be inducible in humans, Dr. Liggins and Dr. (later Professor) Ross Howie, a neonatologist, devised the first randomized controlled trial (RCT) investigating the use of betamethasone for prevention of RDS in preterm infants, which began in 1972 (279). A total of 282 mothers expecting delivery before 37 weeks of gestation were randomized to receive 2 doses of saline or 12 mg betamethasone (6 mg betamethasone phosphate + 6 mg betamethasone acetate, which was the formulation available at the time) given 24 hours apart (279). Maternal betamethasone administration reduced RDS incidence in the babies from 26% to 9% and mortality of infants born before 32 weeks of gestation from 15% to 3% (279). This initial trial provided several crucial insights: infants born 1-7 days after ACS treatment received the greatest benefit, additional ACS courses did not improve outcomes, and the ACS-treated mother and infant had no increased risk of infection compared to the placebo group (279).

#### Evidence synthesis and development of clinical guidelines

Direct comparison between trials was complex, as early trials investigating ACS efficacy greatly differed in design, patient selection, and clinical context. Enrollment was targeted at women at risk of spontaneous preterm labor, but studies varied in the gestational ages studied, and inclusion or exclusion of complications such as preterm premature rupture of membranes (PPROM) and of multiple gestations (279, 288). These early studies, key details of which are described in meta-analyses (18), were collated in the first systematic review of RCTs led by Professor Patricia Crowley in 1990 and highlighted the efficacy of ACS treatment in reducing mortality, RDS incidence and severity, and risks of necrotizing enterocolitis and IVH in preterm babies (289). Subsequent trials, with evaluation and meta-analysis of trial data by organizations such as the UK Cochrane Centre (now developed into the international Cochrane network), provided further evidence of the benefits of ACS treatment (290, 291). After extensive trials demonstrated efficacy, use of ACS was slowly incorporated into clinical practice. Collaborative efforts, such as the 1994 National Institutes of Health consensus conference, played major roles in the development of guidelines recommending the administration of ACS to all women at risk of preterm birth between 24^+0^ and 34^+0^ completed weeks of gestation (292). Increased use of ACS treatment following publication of this recommendation improved respiratory outcomes and decreased mortality in preterm infants (291). There have been subsequent evaluations of ACS use to extend understanding of efficacy in more specific clinical contexts. For example, the immunosuppressive effects of glucocorticoids raised concerns that treatment could increase risk of maternal or neonatal infection in high-risk subgroups, such as women with PPROM. The 1999 Dexiprom trial in South Africa therefore assessed infection outcomes of dexamethasone use in women with PPROM carrying fetuses of 28 to 34 weeks of gestation or unknown gestational age with clinically estimated fetal weight of 1000 g to 2000 g (293). In this study, antenatal dexamethasone did not worsen infection outcomes and improved perinatal mortality outcomes (293). Ongoing research continues to inform revision of clinical practice guidelines.

**Figure 5:**
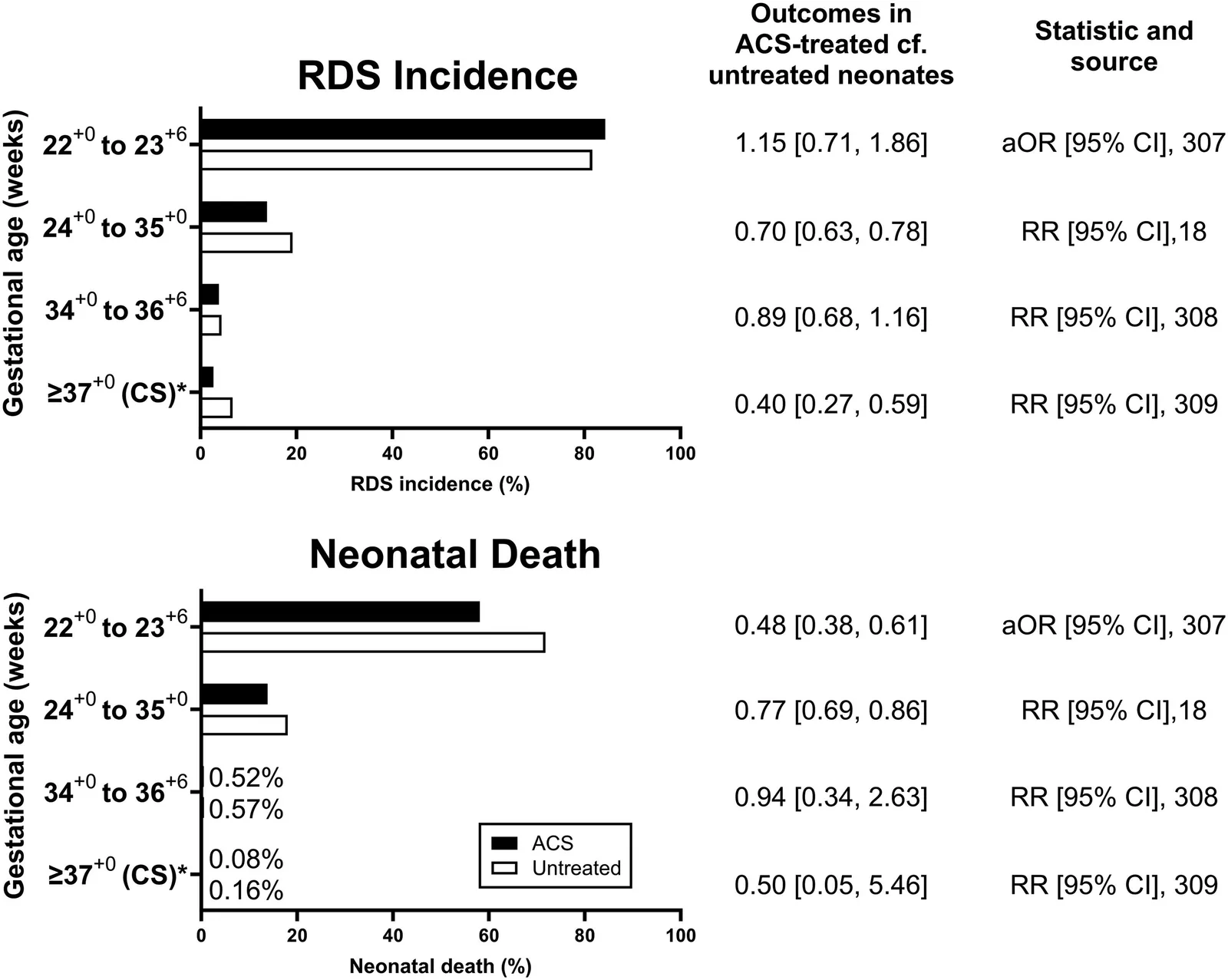
Incidence of respiratory distress syndrome (RDS) and neonatal mortality in preterm infants treated with or without antenatal corticosteroids (ACS) at different gestational ages. The proportion of affected infants are shown for ACS-treated (closed bars) and untreated infants (open bars), with summary statistics from each source indicating the effect of ACS. Data were obtained from systematic reviews of outcomes at each gestational age range, with the majority of included studies from high-resource settings. *Data for term pregnancies delivered at ≥37^+0^ weeks completed gestation are derived from studies of outcomes after planned cesarean section (CS). Abbreviations: ACS, antenatal corticosteroids; aOR, adjusted odds ratio; CS, cesarean section; RDS, respiratory distress syndrome; RR, risk ratio.

Many countries have guideline bodies that work together to create consensus guidelines. For example, the German Society for Gynecology and Obstetrics (DGGG), Austrian Society of Gynecology and Obstetrics (OEGGG), and the Swiss Society for Gynecology and Obstetrics (SGGG) jointly developed the guidelines cited for Germany (Table 2) (13-17, 294-305). Current neonatal mortality rates have declined substantially in countries that have adopted major advancements such as ACS, surfactant therapy, continuous positive airway pressure (CPAP) and mechanical ventilation. In the United States, neonatal mortality rates for babies diagnosed with RDS have been reduced to less than 2% (306). The most recent Cochrane review from 2020 reported that use of ACS reduced risk of RDS by 29% and neonatal death by 22% in babies born at 24^+0^ to 37^+0^ weeks of gestation, with the overall number needed to treat (NNT) being 38 to prevent 1 death (18). There are greater benefits at earlier gestational ages (Fig. 5) (18, 307-309). The NNT to prevent 1 case of RDS, the primary cause of morbidity and mortality in preterm infants, was 55 for infants born at >34 weeks of gestation, but only 19 for infants born between 24 to 35 weeks of gestation (310). Importantly, ACS is an effective antenatal therapy compared to other therapies used before preterm birth, such as magnesium sulfate for fetal neuroprotection which has a NNT of 56 to prevent death or cerebral palsy (311). Glucocorticoid use in the context of preterm birth has similar efficacy for prevention of severe outcomes as use outside the context of pregnancy. The NNT for ACS use before birth at 24^+0^ to 37^+0^ weeks gestation, 19 to prevent severe RDS and 38 to prevent death, are similar to that of glucocorticoids used to treat conditions such as sepsis. Recent analyses determined that the NNT for patients with severe septic shock treated with low-dose hydrocortisone + fludrocortisone was 33 to 35 for 28-day mortality (312) and 17 for 90-day mortality (313).

**Table 2:** Current national recommendations for antenatal corticosteroid use in pregnant women at risk of preterm birth

Region and guideline	Recommended use and eligibility			
	Gestational ages (weeks^+days^)	Eligibility criteria	Steroid type, dose, and timing	Other considerations
Australia and New Zealand Antenatal Corticosteroids Given to Women Prior to Birth to Improve Fetal, Infant, Child and Adult Health: New Zealand and Australian Clinical Practice Guidelines (17)	24^+0^ to 34^+6^	When there is risk of, or plan for, preterm birth within the next 7 days (even if birth is likely within 24 hours), regardless of reason	Single course of 24 mg dexamethasone phosphate (four 6 mg doses given 12 hours apart), OR 24 mg betamethasone sodium phosphate/acetate mix (Celestone® Chronodose®) (two 11.4 mg doses given 24 hours apart)	A repeat dose can be considered if the mother is still at risk of preterm birth within the next 7 days, under 33^+0^ weeks of gestation, and it has been at least 7 days since the last ACS dose
Brazil Acceleration of Fetal Maturity [in Portuguese]*^a^* (294)	24^+0^ to 33^+6^	When there is risk of preterm birth, regardless of reason	Single course of 24 mg betamethasone phosphate/acetate mix (two 12 mg doses given 24 hours apart), OR 24 mg dexamethasone (four 6 mg doses given 12 hours apart)	Includes multiple pregnancies A repeat dose can be considered if the mother is still at risk, under 34^+0^ weeks of gestation, and it has been at least 14 days since the last ACS dose More individualized consideration for women with diabetes
Canada No. 364—Antenatal Corticosteroid Therapy for Improving Neonatal Outcomes (295) Technical Update No. 438: Antenatal Corticosteroids at Late Preterm Gestation (296)	24^+0^ to 33^+6^* *Mothers at 34^+0^ to 36^+6^ weeks of gestation can consider ACS administration based on discussion with healthcare professionals	When there is high risk of preterm birth within 7 days, regardless of reason	Single course of 24 mg betamethasone (two 12 mg doses given 24 hours apart), OR 24 mg dexamethasone (four 6 mg doses given 12 hours apart)	A repeat dose can be considered if the mother is at high risk of preterm birth, under 34^+6^ weeks of gestation, and has only had the first dose^a^ Monitoring of maternal blood glucose recommended after ACS administration ^a^Cancellation of the second dose is considered if preterm delivery risk reduces significantly after the first ACS dose
China Clinical Guidelines for the Prevention and Treatment of Preterm Birth [in Chinese]*^a^* (297)	24^+0^* to 34^+6^	When preterm birth is imminent within the next 7 days, regardless of reason	Single course of 24 mg betamethasone (two 12 mg doses given 24 hours apart, OR four 6 mg doses given 12 hours apart)	A repeat dose can be considered if the mother does not deliver within 7 days, and preterm birth is expected More individualized consideration for mothers with preexisting conditions
Germany Prevention and Therapy of Preterm Birth. Guideline of the DGGG, OEGGG and SGGG (Published English translation of original document [in German]) (Part 1: 298,Part 2: 299)	24^+0^* to 34^+0^ *If neonatal intensive care and maximum medical therapy is available and planned, ACS should be administered if there is threat of preterm birth before 24^+0^ weeks of gestation	When preterm birth is imminent Mothers can be considered low risk if cervical length of >30 mm, or 15-30 mm and negative for fibronectin, phIGFBP-1, and PAMG-1	Single course of 24 mg betamethasone (two 12 mg doses given 24 hours apart), OR 24 mg dexamethasone (four 6 mg doses given 12 hours apart)	A repeat course can be considered if the mother is evaluated to have increasing risk of immediately threatened preterm birth, under 29^+0^ weeks of gestation, and it has been at least 7 days since the previous dose (maximum single course) Mothers between 34^+0^ to 36^+5^ weeks of gestation are advised against ACS administration
India Use of Antenatal Corticosteroids in Preterm Labour (Under Specific Conditions by ANM): Operational Guidelines (300)	24^+0^ to 34^+0^	When preterm birth is imminent, and the mother presents with “true labour pains” (definition includes regular, predictable pain with increasing duration, frequency, and intensity alongside blood-stained mucus discharge)	Single course of 24 mg dexamethasone sodium phosphate (four 6 mg doses given 12 hours apart)	Mothers with diabetes, pre-eclampsia, and hypertension are considered eligible for ACS, although it is advised to monitor blood sugar and pressure
Japan Guidelines for obstetrical practice in Japan: Japan Society of Obstetrics and Gynecology and Japan Association of Obstetricians and Gynecologists 2020 edition (English translation, 301)	24^+0^* to 34^+6^ *Mothers at 22^+0^ to 23^+6^ weeks of gestation can be considered for ACS use depending on individual assessment	When there is high risk of preterm birth within the next 7 days, defined by regular uterine contractions and either progressive cervical dilatation/effacement between 22^+0^ to 36^+6^ weeks of gestation or cervical dilation of >2 cm	Single course of 24 mg betamethasone (two 12 mg doses given 24 hours apart)	More individualized consideration for mothers with preexisting conditions such as carbohydrate metabolism disorders
Malaysia Guidelines on the Prevention and Management of Preterm Birth (302)	24^+0^ to 35^+6^	When preterm birth is imminent, defined by established preterm labor or PPROM	Single course of 12 mg dexamethasone (two 6 mg doses given 24 hours apart)	A repeat course can be considered if the mother is considered still at imminent risk of preterm birth, under 34^+6^ weeks of gestation, and it has been at least 7 days since the last ACS dose (maximum 2 courses) More individualized consideration for mothers with diabetes, such as blood sugar monitoring
Netherlands Threatened Premature Birth [in Dutch] *^a^* (303) Perinatal Policy for Extreme Premature Birth [in Dutch] *^a^* (304)	23^+5^* to 33^+6^** *Mothers at 23^+0^ to 23^+4^ weeks of gestation can be considered for ACS use depending on risk assessment **Mothers at 34^+0^ to 34^+6^ weeks of gestation can be considered for ACS use depending on risk assessment	When preterm birth is imminent within 2-10 days, defined by established preterm labor, PPROM, or cervical dilation of >3 cm	Single course of 24 mg betamethasone sodium phosphate/acetate mix (Celestone® Chronodose®) (two 11.4 mg doses given 24 hours apart)	A repeat course can be considered if the mother is considered at higher risk of preterm birth, under 33^+0^ weeks of gestation, the first ACS course was given before 30^+0^ weeks of gestation, and it has been at least 14 days since the last ACS dose (maximum one course)
Singapore Management of Preterm Labour (305)* *Ministry of Health clinical practice guidelines are considered withdrawn five years after publication unless otherwise specified. Thus, by default, these guidelines are considered withdrawn.	24^+0^ to 35^+6^	When preterm birth is imminent, or when other obstetric factors suggest that delivery should not be delayed	Single course of 24 mg betamethasone (two 12 mg doses given 24 or 12 hours apart) OR dexamethasone (two 12 mg doses given 24 or 12 hours apart)	A repeat dose can be considered if the mother is still considered at risk of preterm birth, although decisions are made on an individual basis More individualized consideration for mothers with severe pre-eclampsia/hypertension
United Kingdom (UK) Green-top Guideline No. 74. (13) NICE Guideline [NG25] (14)	24^+0^* to 34^+6^** *Mothers at 22^+0^ to 23^+6^ weeks of gestation can be considered for ACS use if the conditions are fulfilled and based on individual assessment **Mothers at <38^+6^ weeks of gestation can be considered for ACS use depending on individual assessment and informed discussion about potential risks and benefits	When preterm birth is imminent, defined by established preterm labor or PPROM, or planned preterm birth	Single course of 24 mg betamethasone sodium phosphate/acetate mix (two 12 mg doses given 24 hours apart), OR 24 mg dexamethasone phosphate (two 12 mg doses given 24 hours apart, or four 6 mg doses given 12 hours apart)	A repeat course can be considered if the mother is considered at very high risk of preterm birth in the next 48 hours, under 34^+0^ weeks of gestation, and it has been at least 7 days since the last ACS dose (maximum 2 repeat courses) Suspected fetal growth restriction in mothers less than 30^+0^ weeks of gestation should be considered when considering a repeat ACS course More individualized consideration for mothers with diabetes, such as blood sugar monitoring, but ACS administration is still recommended
United States of America (USA) Committee Opinion No. 713: Antenatal Corticosteroid Therapy for Fetal Maturation (15) Society for Maternal-Fetal Medicine Consult Series #58: Use of antenatal corticosteroids for individuals at risk for late preterm delivery (16)	24^+0^* to 36^+6^ *Mothers from >23^+0^ weeks of gestation can be considered for ACS if the conditions are fulfilled	When there is risk of preterm birth within 7 days, regardless of reason, and based on the family's decision regarding resuscitation	Single course of 24 mg betamethasone (two 12 mg doses given 24 hours apart), OR 24 mg dexamethasone (four 6 mg doses given 12 hours apart)	Includes multiple pregnancies A repeat course should be considered if the mother is still at risk of preterm delivery within 7 days, under 34^+0^ weeks of gestation, and it has been at least 14 days since the last ACS dose (maximum 2 courses)

Abbreviations: ACS, antenatal corticosteroids; PPROM, Premature prelabor rupture of membranes.

^*a*^Guideline with no available English translation, Google Translate used to obtain key information.

#### Comparisons between betamethasone and dexamethasone

Early guideline recommendations favored betamethasone, as some early studies suggested that betamethasone might be associated with lower risk of respiratory conditions in babies born preterm (314, 315). However, the latest Cochrane review (280) reported that betamethasone and dexamethasone similarly reduced relative risks of RDS (RR [95% CI]: 1.06 [0.91-1.22]), chronic lung disease (RR [95% CI]: 0.92 [0.64-1.34]) and neonatal death (RR [95% CI]: 1.03 [0.66-1.63]). Due to limited data availability, it is less clear whether betamethasone and dexamethasone differ in impacts on risks of IVH, necrotizing enterocolitis, and cerebral palsy, or maternal and neonatal infection (280, 316). Some studies report that dexamethasone reduces the incidence of IVH to a greater extent than betamethasone (317) whereas others report similar benefits (316). One retrospective study raised concerns that dexamethasone might induce neurosensory impairment to a greater extent than betamethasone (318). Although the 2019 multicenter A*STEROID trial did not find differences in incidence of neonatal death or neurosensory disability at age 2 following 2 maternal intramuscular injections of 11.4 mg 1:1 betamethasone phosphate + betamethasone acetate or 12 mg dexamethasone sodium phosphate given 24 hours apart (319), further research is warranted. Additional data is required to clarify relative benefits and risks of dexamethasone and betamethasone, and between ACS formulations.

#### Timing of delivery, incomplete courses, and repeat doses

Optimal administration-to-birth intervals for neonatal outcomes are difficult to identify given differences in categorized intervals, ACS regimens, and patient selection across studies. Preterm babies born between 48 hours to 14 days after the first dose have reduced risk of neonatal mortality (320), although only 20% to 40% of babies born <34 weeks of gestation receive ACS within that timeframe (321). For babies delivered <24 hours after the first dose, ACS treatment reduced neonatal mortality in retrospective analysis of large Canadian and European cohorts (322, 323). Nevertheless, mortality benefits were not seen in babies born <24 hours after ACS administration in all studies identified in a recent systematic review, where heterogeneity of design prevented meta-analysis (320). The evidence for reduced risk of RDS when ACS is administered <24 hours before delivery is mixed (320).

The other major question in relation to ACS timing is in relation to persistence of benefit and whether additional doses or courses of ACS should be given. Multiple repeat doses are less recommended and used now than in the past (324), particularly after the second consensus conference in 2000 that highlighted the lack of evidence of benefits and risks of repeat courses (325). Repeat ACS doses in pregnancies where the baby remains at risk of preterm delivery >7 days after the initial ACS course, reduce incidence of RDS and need for ventilator support compared to a single ACS course (326-328). Mortality outcomes are similar in babies exposed to single or repeat ACS treatment (326-328). However, the risk-benefit balance becomes less favorable with increasing ACS exposure. For example, an increasing number of ACS doses is associated with up to 9% lower birthweight and up to 4% lower head circumference in babies born before 33 weeks of gestation (329). Preclinical studies support these findings, with substantial reductions in birthweight following multiple antenatal betamethasone doses in sheep (330). Prolonged ACS exposure may also be detrimental due to its effects on MR signaling. Antenatal betamethasone (331) and dexamethasone (332) suppress cortisol, and therefore cortisol-related MR signaling activity, in mothers and their preterm infants (333). Cortisol suppression inhibits HPA axis responsivity to stressors (334). Reduction of MR activation may have consequences throughout the body, such as reduced MR signaling at certain periods disrupting developmental changes in the brain (335) or heart (176) and alterations in the MR/GR balance increasing the vulnerability of nervous tissue to damage (215). Current clinical guidelines recommend that repeat doses of ACS may be administered to extend lung maturational benefits when preterm birth remains imminent following administration of an initial ACS course 7 to 14 days earlier, depending on country (324). It may be better to delay repeat doses until 14 days after the initial treatment, given that the recent systematic review on timing of treatment-to-birth intervals identified 13 to 14 days post-ACS as the timing with greatest mortality benefits (320).

#### Current global and regional guidelines for antenatal corticosteroid use

The use of ACS has been widely recognized to improve neonatal outcomes and, over the past 3 decades, many countries have adapted their guidelines for managing preterm birth to include ACS treatment (Table 2). Most national protocols refer to evidence-based recommendations by international or multiregional bodies such as the World Health Organization (WHO), International Federation of Gynecology and Obstetrics (FIGO), and European consensus guidelines. However, recommendations for ACS use vary between countries (Table 2) (13-17, 294-305). Some aspects of recommendations are consistent, such as administration by maternal intramuscular injection for most pregnancies and which drugs are used. Betamethasone, the original ACS used in the Liggins trials, and dexamethasone, a cheaper alternative that is widely available, are the most extensively studied and recommended ACS types. The most commonly recommended ACS course (Table 2) (13, 15, 17, 336) consists of 24 mg of ACS comprising either dexamethasone phosphate (four 6 mg doses given 12 hours apart or two 12 mg doses given 24 hours apart) or betamethasone mix of phosphate and acetate (two 12 mg doses given 24 hours apart). The exact formulation used across regions varies greatly. Some regions such as Australia prescribe the 1:1 betamethasone phosphate + betamethasone acetate mix as used in the initial Liggins trial, while UK recommendations are to treat with either the 1:1 betamethasone phosphate + betamethasone acetate mix or dexamethasone phosphate (13, 14, 17). Dexamethasone is often recommended over betamethasone in settings with limited resources due to lower cost. ACS treatment recommendations also vary in gestational age limits, relative timing of doses and use in the presence of various risk factors (Table 2).

Available obstetric guidelines universally recommend ACS use between 24^+0^ and 33^+6^ weeks of gestation (Table 2), with fairly consistent lower gestational age limits for treatment, in contrast to more variable upper gestational age limits. Some high-resource countries, such as Japan and UK, extend the lower gestational age limit for eligibility to 22^+0^ weeks of gestation based on individual assessment as they have the infrastructure required to support extremely preterm babies. Most countries only recommend use in pregnancies at 24^+0^ weeks of gestation or later (Table 2). ACS use for late preterm infants remains controversial, with inconsistent recommendations (Table 2), which in part reflect clinical concerns about off-target effects of ACS on neurodevelopment (337, 338). Guidelines generally cover singleton pregnancies or do not specify plurality, although some countries such as Brazil and the United States recommend that pregnancies with single and multiple fetuses are treated similarly (15, 294). All guidelines recommend caution with repeat doses due to diminished benefits and potential fetal growth deficits. Nevertheless, the latest Cochrane review of published evidence reported neither short-term benefit nor significant harm of repeat ACS doses to parent or child (324). In cases with preexisting maternal conditions, such as diabetes mellitus, clinicians are advised to take additional precautions, such as blood glucose monitoring, but clinical guidelines cite insufficient evidence to make recommendations for or against ACS use within these groups. Many trials have excluded subjects with these preexisting maternal conditions due to concerns regarding the potential impact of ACS on glycemic control (339, 340), and more studies are required to investigate safety and efficacy of ACS use in these populations. In addition to the variability in eligibility, there are concerns regarding ACS use in low- and middle-resource locations due to issues such as feasibility and access to NICU (341). The use of ACS is common in high-resource countries, with 69.4% of preterm infants in the United States treated with ACS, but sporadic in low- and middle-resource countries (342). Recent evidence of ACS benefit in low-resource settings is reassuring, with any number of dexamethasone doses reducing neonatal mortality by 36.7% and a complete course reducing neonatal mortality by 41.6% in settings without ventilatory support, compared to preterm infants whose mothers received no steroids (343). Additional trials assessing ACS efficacy and safety in low-resource regions are currently underway (344).

## Individual factors affecting responsiveness to antenatal corticosteroids

### Introduction

A diverse spectrum of responses to ACS is observed in preterm infants (18, 20). Some infants are not responsive to prenatal exposure to glucocorticoids and remain intubated and oxygen-dependent after ACS treatment (18, 345). These individuals are often designated as “nonresponders” in other species. In ACS-treated preterm rabbits, 25.5% of kittens have static lung pressure-volume relationships comparable to untreated controls (346, 347). In preterm sheep, 38.6% of newborn lambs exposed to maternal ACS treatment have similar lung function as untreated controls (348). There are multiple factors implicated in variable responsiveness to ACS, including gestational age, fetal sex, fetal growth restriction, and pregnancy complications (310).

### Gestational age

Before 22^+0^ weeks of gestation, during the early canalicular stage of lung development, human fetal lungs are considered insufficiently developed to support effective GR-mediated responses (307). This is supported by preclinical evidence. For example, lung GR abundance increases with gestational age in the rabbit (349), and in sheep, ACS increases surfactant expression in the saccular stage of lung development (350, 351) but not earlier in gestation during the canalicular stage (352, 353). ACS use in extremely preterm babies is controversial, with evaluation of benefit complicated by variation in the gestational ages included in different studies. Meta-analyses of data for babies born <25^+0^ weeks of gestation shows reduced risk of death following ACS in the combined cohort and within 22-, 23-, and 24-week-old subgroups (354). An earlier meta-analysis that included RDS as well as mortality outcomes for babies born at 22^+0^ to 23^+6^ weeks of gestation, reported that ACS reduced risk of death but not RDS overall (Fig. 5) (307). In contrast to these early preterm groups, there is clear evidence that ACS reduces risks of RDS and mortality in babies born at 24^+0^ to 35^+0^ weeks of gestation (Fig. 5) (18).

The lungs of late preterm fetuses at 34^+0^ to 36^+0^ weeks of gestation are relatively mature, with abundant surfactant production, limiting potential benefits of ACS (71). This likely explains the lack of ACS benefit for RDS or mortality seen in meta-analysis of data for late preterm infants (Fig. 5) (308). However, clinical uncertainty about ACS treatment of late preterm infants remains and is reflected in variable upper gestational age limits in clinical guidelines (Table 2). Early trials, such as the Brazilian single-center RCT study led by Porto and coauthors did not show benefit of maternal betamethasone for neonatal respiratory outcomes (adjusted risk [95% CI]: 1.12 [0.74-1.70]) (355). Clinical guidelines in the United States changed to recommend ACS use in late preterm populations following the 2016 Antenatal Late Preterm Steroid (ALPS) trial (356), an American multicenter RCT in which betamethasone treatment at 34^+0^ to 36^+5^ weeks of gestation reduced severe neonatal respiratory complications (RR [95% CI]: 0.66 [0.52-1.82]). Betamethasone treatment reduced need for respiratory support and neonatal mortality without increase in neonatal sepsis or chorioamnionitis (356). Late preterm ACS use remains under consideration in other regions. The recent WHO ACTION-II trial supported a potential benefit of dexamethasone use in late preterm populations in low-resource countries (357), and further study is ongoing in the WHO ACTION-III trial (344).

Given the lack of benefit for ACS treatment of late preterm infants reported in meta-analysis (Fig. 5) (308), it is unlikely that ACS treatment would benefit term populations overall. However, impacts of ACS have been assessed in term populations that have above-average incidence of respiratory problems at birth. For example, babies born following elective cesarean section, and not exposed to labor, are at increased risk for developing neonatal respiratory complications (358). In term babies delivered by elective cesarean section, ACS treatment was associated with reduced risk of RDS, but unchanged risk of mortality (Fig. 5) (309). Whether ACS reduces risks of RDS or mortality in babies born at term vaginally is unclear, although given low prevalence of RDS at term overall (358), it is unlikely that benefits of ACS would outweigh potential risks, such as for neurodevelopment (359).

### Fetal sex

Among preterm neonates, males consistently have worse pulmonary and neurological outcomes than females born at the same gestational age (238, 360-364). Without ACS treatment, preterm males have an ∼1.7-fold higher RDS incidence than preterm females (237, 238). The higher incidence of respiratory morbidity in males becomes more marked with decreasing gestational age, with the disparity most evident in extremely preterm infants (365, 366). There is currently clinical uncertainty about the sex-specific benefits of ACS as many studies and the largest meta-analysis of ACS efficacy (18) do not split outcomes by fetal sex. In an early study of maternal betamethasone treatment at 27 to 34 weeks gestation (course of 2 injections with 12 mg betamethasone, formulation not stated), the authors reported greater benefit in females than males (238). This was based on a shift in the ratio of males to females with RDS at the same hospitals from 1.7:1 in untreated babies (pre-betamethasone introduction, 1973-1975) to 3.4:1 in betamethasone-treated babies (born 1977-1979) (238). In contrast, in a recent reanalysis of the original Liggins (Auckland) trial conducted in 1969-1974 (367), subgroup analyses indicated greater efficacy of betamethasone in male than female infants, with adjusted risk ratios for RDS of 0.43 (95% CI 0.28-0.67) in boys and 1.01 (95% CI 0.61-1.69) in girls. Furthermore, sex differences in benefits of ACS may differ between steroids. In a meta-analysis of 8 eligible studies that reported sex-specific outcomes (368), risks of RDS were reduced similarly (*P* = .99) in ACS-exposed than control babies of both sexes overall (RR 0.50 [95% CI 0.33-0.77] for males, and RR 0.57 [95% CI 0.43-0.75] for females). However, in subgroup analyses split by ACS type, betamethasone treatment (RR 0.29 [95% CI 0.15-0.57]), but not dexamethasone (RR 0.78 [95% CI 0.57-1.07]) reduced risk of RDS in males (368). Conversely, dexamethasone treatment (RR 0.51 [95% CI 0.32-0.81]), but not betamethasone (RR 0.62 [95% CI 0.38-1.00]), reduced risks of RDS in female infants (368). These results are consistent with those of the original Auckland trial (367), which was not included in the meta-analysis, and suggest that ACS efficacy may differ between sexes depending on the ACS used. Additional analysis of outcomes in contemporary cohorts and/or individual patient data meta-analysis from additional trials would be useful to further test this hypothesis.

Males have delayed fetal lung maturation compared to females, which can be partially attributed to their sex hormones (369-371). Androgens such as testosterone and 5α-dihydrotestosterone (5α-DHT) inhibit AECII maturation and surfactant production, exerting effects that are opposite to glucocorticoids. Moreover, glucocorticoid actions can be decreased through 5α-DHT administration, which reduces lung GR mRNA and protein expression (372). Although several studies have investigated sex-related differences in the abundance of surfactants and lung compliance following preterm birth (373, 374), few have examined these outcomes in the context of sex-specific differences in ACS response (368). Some preclinical studies report that lung physiology after ACS treatment differs between sexes. The increase in surfactant production after betamethasone exposure, relative to sex-matched controls, was lower in male rabbit fetuses compared to females (375, 376). Betamethasone treatment also induced a greater improvement in airspace volume fraction and better deflation stability in preterm female rabbits than males (377). These findings were similarly reflected in near term lambs, where betamethasone improved tidal volume, dynamic lung compliance, and oxygenation more in females than males (351). The molecular mechanisms underlying sex-specific ACS responses remain unclear and there is insufficient evidence to assess whether the difference in response merits differing treatment strategies for preterm human pregnancies according to fetal sex.

Sex differences in ACS response are also influenced by the sex differences in placental response to environmental signals, which impact neonatal adrenal function. In utero, females are more sensitive than males to elevated glucocorticoid concentrations, reducing growth through alterations in placental GR expression and fetal HPA axis function to increase chances of survival (20, 378, 379). Conversely, males adjust adrenal function to sustain continued growth in response to glucocorticoid exposure (20, 379, 380). In human preterm infants treated with antenatal betamethasone, females had both higher placental 11β-HSD2 activity and higher urinary cortisol excretion than males (20), implying that there are sex-specific differences in both placental and fetal responses to betamethasone in preterm babies. Similar differences in placental gene expression have also been reported in animals treated with ACS near term. In near term sheep placentae, betamethasone induces expression of genes involved in oxidative stress and apoptosis to a greater extent in females than in males (381). In mice, repeated maternal dexamethasone treatment at mid-pregnancy upregulated gene expression of multiple pro-inflammatory genes, chemokines, and receptors in male placentas while the majority of those genes were downregulated in female placentas (382). Although this study provides evidence of sex-specific placental responses to ACS, the doses used (1 mg/kg, daily for 3 days) are higher than commonly used to induce fetal lung maturation in rodents. Whether ACS treatment should be customized based on fetal sex is a question requiring further research.

Differential expression of GR isoforms in tissues may contribute to sex differences in fetal responsiveness to glucocorticoids (25). Total GR number and glucocorticoid binding affinity did not differ between sexes in fetal sheep exposed to maternal dexamethasone treatment (383), or in cultures of lung cells from fetal rabbits (384) and rats (385) treated with dexamethasone, suggesting that poor male response to ACS may be mediated by other factors. Lower glucocorticoid sensitivity in males is associated with lower GRα translocation and higher expression of GRβ (386). GRβ is a known GRα antagonist that can inhibit GRα activation in response to glucocorticoids and also associated with pro-inflammatory mediators such as TNFα and interferon-γ (IFN-γ) (52). Human male placentae have a higher cytoplasmic GRβ: GRα ratio compared to female placentae, which is linked to lower glucocorticoid-induced apoptosis (52, 387). In a preclinical rat study, the placental extracts of preterm males with dexamethasone treatment had increased cytoplasmic expression of GRα-A, involved in stress and immune response and metabolism, and decreased cytoplasmic expression of GRα-C, involved in glucocorticoid-induced apoptosis, compared to dexamethasone-treated preterm female placentae (388). However, in humans, the only GR isoform with a sex-specific difference was GRα-D2, with betamethasone treatment increasing nuclear expression in preterm male placentae relative to preterm females (22). Greater nuclear localization of GRβ, GR-A, and GR-P in male placentae compared to females has also been reported in pregnancies complicated by chronic respiratory conditions such as asthma (389). The higher nuclear expression of pro-apoptotic GR isoforms in females may allow the female placentae to maintain greater sensitivity to glucocorticoids, allowing greater adrenal adaptation to stress and improved chances of survival (381).

### Fetal growth restriction

Fetal growth restriction (FGR), in which fetal growth does not meet the genetic potential for growth, often due to insufficient nutrient supply, is associated with increased risk of neonatal morbidity and mortality (390, 391). There are many potential causes of FGR, although placental insufficiency and maternal undernutrition are the most common (392). FGR, previously known as intrauterine growth restriction (IUGR), had variable definitions, leading to major challenges in detection, prevention, and treatment for growth-restricted fetuses (393). The current consensus definition of FGR is split into early and late FGR (394). Early FGR (<32 weeks) needs to satisfy 1 of 3 solitary parameters (abdominal circumference [AC] < 3rd centile, estimated fetal weight [EFW] < 3rd centile and absent end-diastolic flow in the umbilical artery [UA]) or several contributory parameters (AC or EFW < 10th centile with UA or uterine artery pulsatility index [PI] of >95th centile). Late FGR (≥ 32 weeks) needs to satisfy either solitary parameters (AC or EFW <3rd centile) or several contributory parameters (AC or EFW <10th centile, AC or EFW crossing centiles by > 2 quartiles on growth charts and cerebroplacental ratio <5th centile or UA-PI >95th centile). International bodies such as International Federation of Gynecology and Obstetrics and the International Society of Ultrasound in Obstetrics and Gynecology (ISUOG), as well as some countries such as the United States and Australia, have adopted small for gestational age (SGA), defined as birthweight less than the 10th centile for gestational age, as a proxy for FGR (395-398). Babies born SGA account for 1 in 5 live births (399). However, SGA does not account for individualized growth potential of each fetus, so some SGA fetuses can be constitutively healthy but small due to their genetic growth potential. Moreover, some growth-restricted fetuses may remain undetected despite not meeting their genetic growth potential, since they are above the SGA percentile threshold based on current clinical weight estimation methods for fetuses, such as sonographic detection, which has a high error margin (397). In preclinical and clinical studies, the terms “*FGR*” and “*SGA*” may be defined differently or sometimes even used interchangeably (393). These inconsistent definitions complicate comparisons between studies (400).

Preterm birth is a common complication associated with FGR. Globally, 6% of preterm infants are born SGA whereas 4.2% of term-born infants are born SGA (401). The effect of FGR on prenatal lung development remains controversial (402). Relative to normal-growth counterparts, infants with early-onset FGR have lower surfactant production and lung maturation (403), whereas late-onset FGR infants have higher surfactant production and blood oxygenation (403-406), and the detrimental impact on respiratory function may be further exacerbated by preterm birth (407). There is some evidence that FGR-induced impairments persist to adulthood, with poorer airway function (408) and structural alterations in the lung such as fewer alveoli and greater interalveolar septae thickness (407, 409). In contrast, others report that FGR does not alter surfactant expression, lung liquid clearance, or lung development (410), and that FGR-induced structural impairments are no longer evident after 8 weeks postnatal age (411). Recent systematic reviews report an association between FGR and poorer lung function in humans, although it is noted that most studies defined groups based on SGA and low birthweight rather than FGR (412).

Most current clinical guidelines recommend ACS use in preterm infants regardless of their growth status. However, while ACS improves preterm outcomes in the overall population (18), they seem to confer limited benefits for FGR infants. In a systematic review published in 2016, most studies reported no benefit of ACS for RDS incidence in SGA or FGR infants, although a meta-analysis was not performed due to the heterogeneous definitions of FGR (400). More recently, in a retrospective Canadian cohort study of >6500 neonates, the reductions in neonatal mortality and need for mechanical ventilation in response to ACS treatment were similar in individuals born SGA and their appropriately grown counterparts (413). A recent narrative review highlights limited data and mixed evidence of ACS benefit in early FGR populations and a lack of benefit of ACS for neonatal RDS, morbidity, and mortality in pregnancies complicated by late FGR, based on 2 studies (414). Given this variation in outcomes, developers of clinical guidelines are hesitant to recommend for or against ACS use in the context of FGR, although preclinical studies are helping to breach the gap in understanding and inform policies. A recent meta-analysis of preclinical evidence suggests that the effects of ACS are similar in preterm SGA fetuses compared to their preterm appropriately growth counterparts, with improved fetal lung maturation, similar effects on body and brain weight, and reduced mortality (415). However, only 8 studies were included in that systematic review.

Several biological mechanisms might contribute to decreased ACS responsiveness in FGR, including reduced placental glucocorticoid barrier function, greater pretreatment fetal exposure to endogenous glucocorticoid and altered GR isoform expression. In FGR pregnancies, the placenta has lower expression of 11β-HSD2 (416-418), which converts active glucocorticoids into their inactive form, and lower activity of placental P-glycoprotein (281, 419), which can shuttle glucocorticoids out of cells and avoid activating GR signaling. Thus, the ability to inhibit active glucocorticoids from entering the fetal compartment is compromised in FGR fetuses, and FGR fetuses have elevated circulating cortisol concentrations in earlier stages of development (391). This exposure to excess circulating glucocorticoids is predicted to reduce responsiveness to glucocorticoids later in gestation through negative feedback on GR expression. Differential expression of GR isoforms may also contribute to poorer ACS responsiveness, since FGR fetuses have greater lung expression of GR isoforms linked to glucocorticoid-induced apoptosis (420). Fetal sheep with FGR induced by maternal nutrient restriction had lower lung expression of GRα, GRβ, and surfactant proteins and elevated cortisol concentrations in the circulation at near term and term ages compared to controls (420-422). Differences in GR isoform expression in SGA placentae has also been seen in human pregnancies. Male preterm placental GR expression did not differ based on betamethasone exposure or birthweight, whereas SGA preterm female placentae had lower expression of nuclear GR-A, nuclear GRα-D2, and cytoplasmic GRα-D1 compared to their appropriately growth counterparts (22). These 2 mechanisms may be related, since elevated circulating cortisol concentrations are associated with higher cytoplasmic and nuclear GRβ expression in male SGA placentae compared to female SGA placentae (389). In a preterm sheep model, in which lambs were delivered 48 hours after maternal intramuscular injection with 1:1 betamethasone acetate + betamethasone phosphate mix, neither maternal nor fetal circulating concentrations of betamethasone differed between responders and nonresponders defined based on ventilation outcomes (348). This is consistent with the hypothesis that treatment benefit depends on intrinsic fetal responsiveness, rather than differing magnitude of ACS exposure, at least within pregnancies given the same steroid and dose regimen.

### Pregnancy complications

#### Diabetes in pregnancy

Gestational diabetes mellitus (GDM), defined as glucose intolerance with onset or first recognition during pregnancy (423), is a common pregnancy complication associated with increased risks of preterm birth and RDS (424). The latter is likely due to increased transfer of glucose to the fetus, as hyperglycemia delays lung maturation by interfering with surfactant lipid and protein synthesis, decreasing alveolar surface tension (425). Data on impacts of ACS in pregnancies complicated by diabetes are limited, with low certainty evidence from a single study that ACS therapy increases risk of infant NICU admission (426). Effects of ACS on risks of mortality and RDS in this population are very uncertain (426). Several clinical guidelines recommend that ACS is not used in the presence of maternal diabetes (Table 2). Administration of ACS increases circulating glucose concentrations in pregnant women regardless of preexisting glycemic status, and women with preexisting diabetes or gestational diabetes mellitus have a greater incidence of severe hyperglycemia than those who were normoglycemic before treatment (427, 428). Direct fetal intramuscular injection with ACS can be used clinically in pregnancies complicated by diabetes to avoid maternal hyperglycemia, but there are very limited data on health outcomes (429).

#### Chorioamnionitis

Like diabetes, pregnancies complicated by chorioamnionitis are at increased risk of preterm delivery (430). In contrast to diabetes, however, the presence of histological chorioamnionitis is associated with a reduced rather than increased risk of RDS (430). This is supported by preclinical evidence in preterm sheep, where intraamniotic administration of lipopolysaccharide, mimicking signals of intrauterine infection, induces robust lung maturation, including surfactant expression (256). Despite lung maturational effects of chorioamnionitis, the available low certainty evidence is that ACS use reduces the likelihood of neonatal death (pooled OR: 0.51 [95% CI 0.31-0.85]), IVH (pooled OR: 0.41 [95% CI 0.23-0.72]), and RDS (pooled OR: 0.59 [95% CI 0.45-0.77]) in women with chorioamnionitis (431).

## Conclusions

Glucocorticoid exposure in late gestation is crucial for preparing the fetus for extrauterine life, inducing changes in gene and protein expression that subsequently promote organ development and systemic function. Increasing exposure of fetal tissues to endogenous glucocorticoids in late gestation is achieved by species-specific mechanisms, varying in whether the main source is fetal or maternal. ACS accelerate lung maturation and alleviate the risk of respiratory disease in preterm infants, although neonatal responsiveness and benefits of ACS treatment vary. Clinical guidelines consistently recommend maternal intramuscular injection of a course of betamethasone or dexamethasone where there is a risk of preterm birth between 24^+0^ and 33^+6^ weeks of gestation, with variation in recommendations for older gestational ages and in the presence of pregnancy complications. Studies of ACS have used different inclusion criteria, including gestational age ranges, and variable definitions for different conditions, such as FGR and RDS, that complicate synthesis of outcomes. Further research is needed to compare the maturational benefits of ACS in preterm infants in pregnancies complicated by FGR, to assess potential interactions between ACS type and fetal sex, and to explore mechanisms underlying variable responsiveness to ACS.

## Abbreviations

11β-HSD: 11β-hydroxysteroid dehydrogenase
ACS: antenatal corticosteroids
ACTH: adrenocorticotropic hormone
AEC: alveolar epithelial cell
AECI/II: alveolar epithelial cells type I/II
AP-1: activator protein-1
AVP: arginine vasopressin
CBG: corticosteroid-binding globulin
CRH: corticotropin-releasing hormone
eNaC: epithelial sodium channel
FGR: fetal growth restriction
GI: gastrointestinal
GR: glucocorticoid receptor
GRE: glucocorticoid response element
HPA: hypothalamic-pituitary-adrenal
IVH: intraventricular hemorrhage
LBD: ligand-binding domain
MR: mineralocorticoid receptor
NF-κB: nuclear factor-κB
NICU: neonatal intensive care unit
NNT: number needed to treat
RCT: randomized controlled trial
RDS: respiratory distress syndrome
RR: relative risk
SGA: small for gestational age
WHO: World Health Organization
